# sPLA2-IIA modifies progranulin deficiency phenotypes in mouse models

**DOI:** 10.1186/s13024-025-00863-8

**Published:** 2025-06-17

**Authors:** Cha Yang, Huan Du, Gwang Bin Lee, Masaaki Uematsu, Weiguo He, Etienne Doré, Weizhi Yu, Ethan J. Sanford, Marcus B. Smolka, Eric Boilard, Jeremy M. Baskin, Ling Hao, Fenghua Hu

**Affiliations:** 1 Department of Molecular Biology and Genetics, 345 Weill Hall, Ithaca, NY 14853 USA; 2 Department of Chemistry and Chemical Biology, Ithaca, NY 14853 USA; 3 Weill Institute for Cell and Molecular Biology, Cornell University, Ithaca, NY 14853 USA; 4 Centre de Recherche du Centre Hospitalier Universitaire de Québec, Centre de Recherche ARThrite - Arthrite, Recherche, Traitements, Université Laval, Québec, QC Canada; 5 Department of Chemistry and Biochemistry, University of Maryland, College Park, MD 20742 USA

**Keywords:** Progranulin, Inflammation, Lysosome, Mouse strain background, sPLA2-IIA, Mitochondria

## Abstract

**Background:**

Haploinsufficiency of the progranulin (PGRN) protein is a leading cause of frontotemporal lobar degeneration (FTLD). Mouse models have been developed to study PGRN functions. However, PGRN deficiency in the commonly used C57BL/6 mouse strain background leads to very mild phenotypes, and pathways regulating PGRN deficiency phenotypes remain to be elucidated.

**Methods:**

We generated PGRN-deficient mice in the FVB/N background and compared PGRN deficiency phenotypes between C57BL/6 and FVB/N backgrounds via immunostaining, western blot, RNA-seq, and proteomics approaches. We demonstrated a novel pathway in modifying PGRN deficiency phenotypes using inhibitor treatment and AAV-mediated overexpression in mouse models.

**Results:**

We report that PGRN loss in the FVB/N mouse strain results in earlier onset and stronger FTLD-related and lysosome-related phenotypes. We found that PGRN interacts with sPLA2-IIA, a member of the secreted phospholipase A2 (sPLA2) family member and a key regulator of inflammation, that is expressed in FVB/N but not C57BL/6 background. sPLA2-IIA inhibition rescues PGRN deficiency phenotypes, while sPLA2-IIA overexpression drives enhanced gliosis and lipofuscin accumulation in PGRN-deficient mice. Additionally, RNA-seq and proteomics analysis revealed that mitochondrial pathways are upregulated in the PGRN-deficient C57BL/6 mice but not in the FVB/N mice.

**Conclusions:**

Our studies establish a better mouse model for FTLD-*GRN* and uncover novel pathways modifying PGRN deficiency phenotypes.

**Supplementary Information:**

The online version contains supplementary material available at 10.1186/s13024-025-00863-8.

## Introduction

Progranulin (PGRN), encoded by the *granulin (GRN)* gene in humans, has emerged as a key player in brain aging and neurodegenerative diseases [1, 2]. Mutations in the *GRN* gene, resulting in PGRN haploinsufficiency, are one of the major causes of frontotemporal lobar degeneration (FTLD) with TDP-43 positive inclusions [3–5]. Complete loss of PGRN in humans causes neuronal ceroid lipofuscinosis (NCL) [6–8], a group of lysosomal storage diseases (LSDs). *GRN* was also identified as one of the two main determinants of differential aging in the cerebral cortex [9] and as one of the five risk factors for limbic-predominant age-related TDP-43 encephalopathy (LATE) [10]. Polymorphisms in the *GRN* gene also contribute to the risk of Alzheimer’s disease (AD) [11–14], and *GRN* is closely linked to Parkinson’s disease (PD) [15, 16].

PGRN is an evolutionarily conserved glycoprotein comprised of 7.5 granulin repeats with critical roles in inflammatory responses [17–20]. PGRN is highly expressed in microglia, the resident immune cell in the brain, and regulates many aspects of microglial behavior [20]. PGRN deficiency results in exacerbated microglial activation, enhanced phagocytosis, increased levels of complement factors, and excessive synaptic pruning, leading to the disruption of brain circuits [21]. At the cellular level, PGRN is either secreted to the extracellular space or sorted into the lysosomal compartment within the cell [2, 22]. PGRN is processed into granulin peptides in the lysosome and is critical for proper lysosomal functions, especially during aging [2, 22]. Despite these studies, the precise mechanisms by which PGRN regulates brain inflammation and lysosomal functions remain elusive.

It is well established that the genetic background of mouse strains influences disease-associated phenotypes [23–26]. Various mouse strains, such as C57BL/6 and BALB/c, have been used to explore the genetic and phenotypic variability in the manifestation of neurodegenerative diseases, including multiple LSDs and AD [27–29]. Commonly used PGRN knockout (*Grn*^*−/−*^) mouse lines in the C57BL/6 (hereafter B6) background are known to develop gliosis and lysosomal abnormalities in an age-dependent manner, with mild glia activation and lipofuscin accumulation typically observed by 7 months of age [30]. In addition, no detectable or very mild TDP-43 pathology is observed in aged *Grn*^*−/−*^ mice in this background [30, 31]. In this study, we report that PGRN deficiency in the FVB/N (hereafter FVB) background leads to earlier and more severe phenotypes compared to the B6 background, including elevated microglia and astrocytes activation, lysosomal defects, and TDP-43 pathology. To dissect the mechanisms behind the strain background differences, we investigate the role of sPLA2-IIA, a secreted phospholipase found to interact with PGRN in a proteomic screen. Interestingly, sPLA-IIA is expressed in FVB but not B6 strains. We showed that sPLA2-IIA activities and levels are downregulated by PGRN. Furthermore, sPLA2 inhibitors rescue PGRN deficiency phenotypes in the FVB mice, and AAV-mediated sPLA2-IIA overexpression leads to enhanced phenotypes in *Grn*^*−/−*^ mice in the B6 background. To further explore the mechanisms behind the strain background differences affecting phenotypes associated with PGRN deficiency, we performed RNA-seq and proteomics analyses and found pronounced transcriptome and proteome differences between the FVB and B6 strains. Intriguingly, the mitochondrial pathways are differentially regulated by PGRN deficiency in the two backgrounds, which might affect PGRN deficiency phenotypes. Taken together, our studies establish a better model to study PGRN function in mice and identify novel pathways regulating PGRN deficiency phenotypes.

## Materials and methods

### DNA and plasmids

Human *Pla2g2a* cDNA in the pDONR221 vector was obtained from DNASU. Human *Pla2g2a*-Myc-His construct was obtained through a gateway reaction with the pDONR221-*Pla2g2a* entry vector and an engineered destination vector with CMV promoter and Myc-His tag (h-sPLA2-IIA-myc). Human PGRN construct was previously described [32, 33]. Mouse *Pla2g2a*-Myc-DDK construct was obtained from Origene (m-sPLA2-IIA-myc). Mouse PGRN construct was cloned into pSecTag2B vector (Invitrogen) with an N-terminal FLAG-His tag (Flag-mPGRN).

### Primary antibodies and reagents

The following antibodies were used in this study: mouse anti-Myc (9E10) (Sigma, 1:1000 for western blot), mouse anti-GAPDH (Proteintech Group, 60004-1-Ig, 1:10000 for western blot), mouse anti-actin (Proteintech Group, 66009-1-Ig, 1:5000 for western blot), rat anti-mouse LAMP1 (1D4B) (BD Biosciences, 553793, 1:300 for immunostaining), sheep anti-mouse PGRN (R&D systems, AF2557, 1:1000 for western blot and 1:100 for immunostaining), goat anti-CathD (R&D systems, AF1029, 1:1000 for werstern blot and 1:100 for immunostaining), sheep anti-mouse sPLA2-IIA (ThermoFisher Scientific, PA5-47672, 1:1000 for western blot), mouse anti-NeuN (Millipore, MAB377, 1:500 for immunostaining), rabbit anti-IBA-1 (Wako, 01919741, 1:500 for immunostaining), rat anti-CD68 (Bio-Rad, MCA1957, 1:300 for immunostaining), mouse anti-GFAP (Cell signaling, 3670 S, 1:3000 for western blot and 1:500 for immunostaining), mouse anti-galectin-3 (BioLegend, 126702, 1:1000 for western blot and 1:300 for immunostaining), goat anti-GPNMB (R&D Systems, AF2330, 1:300 for immunistaining), rabbit anti-TDP43 (Proteintech Group, 12892-1-AP, 1:5000 for western blot), and rabbit anti-phospho-TDP-43 (Ser409/410) (Proteintech group, 80007-1-RR, 1:5000 for western blot). Myc-Trap (ChromoTek) and GFP-trap (Proteintech Group) were used for anti-myc and anti-GFP immunoprecipitations, respectively.

The following reagents were also used in the study: Dulbecco’s modified Eagle’s medium (DMEM) (Cellgro, 10-017-CV), 0.25% Trypsin (Corning, 25-053-CI), LY333013 (Chemietek, CT-VAREM), KH064 (Millipore Sigma, S3319), Odyssey blocking buffer (LI-COR Biosciences, 927-40000), protease inhibitor (Roche, 05056489001), Pierce BCA Protein Assay Kit (Thermo Scientific, 23225), O.C.T compound (Electron Microscopy Sciences, 62550-01), mouse sPLA2 activity assay Kit (Cayman Chemical, 765001).

### Mouse strains and treatments

*Grn*^*−/−*^ mice in the B6 background [31] mice were obtained from Jackson Labs and housed in the Weill Hall animal facility. To generate the *Grn*^*−/−*^ mice in the FVB background, male B6 *Grn*^*−/−*^ mice (*Pla2g2a*^*−/−*^*Grn*^*−/−*^) were mated to female WT FVB (*Pla2g2a*^*+/+*^*Grn*^*+/+*^) mice to generate *Pla2g2a*^*+/−*^*Grn*^*+/−*^ mice. *Pla2g2a*^*+/−*^*Grn*^*+/−*^ mice were bred with each other to generate *Pla2g2a*^*+/+*^*Grn*^*−/−*^ mice in the FVB/B6 background. An additional 6 backcrosses with female wild-type FVB were made to generate *Grn*^*+/−*^ mice in a pure FVB background. To generate *Grn*^*−/−*^ mice with wild type *Pla2g2a* expression on a B6 background, FVB *Grn*^*−/−*^ mice (*Pla2g2a*^*+/+*^*Grn*^*−/−*^) were mated to female B6 *Grn*^*−/−*^ mice (*Pla2g2a*^*−/−*^*Grn*^*−/−*^) to generate *Pla2g2a*^*+/−*^*Grn*^*−/−*^ mice. *Pla2g2a*^*+/−*^*Grn*^*−/−*^ mice were bred with B6 mice for an additional 6 backcrosses to get these mice with a pure B6 background. *Pla2g2a* genotyping was performed using the following primers: Forward common, 5’- CAGAGCTGACAGCATGAAGGTCCTC-3’; Reverse WT, 5’-TCTGTGGCATCCTTGGGGGAT-3’; Reverse *pla2g2a* knockout, 5’- CTGTGGCATCCTTGGGGGAA-3’.

For inhibitor treatment, 5-week-old *Pla2g2a*^*+/+*^*Grn*^*−/−*^ mice were fed with 0.3% DMSO in corn oil, LY333013 (10 mg/kg, dissolved in DMSO and diluted in corn oil) or KH064 (10 mg/kg, dissolved in DMSO and diluted in corn oil) via oral gavage once per day for 3 consecutive weeks. Both male and female mice were included in each group. The age of the mice used for each experiment is described in the figure legend. All the mice were housed in the Weill Hall animal facility at Cornell. All animal procedures have been approved by the Institutional Animal Care and Use Committee (IACUC) at Cornell.

### Adeno-associated virus (AAV) production and injection

The AAV-*Pla2g2a* transgene (pAAV-m*Pla2g2a*) was generated by cloning mouse *pla2g2a* cDNA into a single-stranded AAV vector containing a chicken β-actin (CBA) promoter and a bovine growth hormone polyadenylation (BGH-PolyA) signal. A Kozak sequence was added for efficient initiation of translation, and the woodchuck hepatitis virus posttranscriptional regulatory element (WPRE) was incorporated to enhance sPLA2-IIA expression. The ssAAV-*Pla2g2a* was packaged into AAV9 capsids, and the virus was purified and titrated using a q-PCR-based approach by VectorBuilder. For AAV injection in mice, newborn WT and *Grn*^*−/−*^ C57BL/6 mouse pups (P0) were intravenously injected with 4 µL of AAV9-m*Pla2g2a* (1.47 × 10^13^ GC/mL) or the control buffer (10 mM Phosphate, 138 mM NaCl, 2.7 mM KCl, 0.001% Pluronic-68) via retro-orbital route as described previously [34]. Mice were collected at 2 months old.

### Cell culture and DNA transfection

HEK293T cells were maintained in Dulbecco’s Modified Eagle’s Medium (Cellgro) supplemented with 10% fetal bovine serum (Sigma) in a humidified incubator at 37ºC with 5% CO2. For transient overexpression, HEK293T Cells were transfected with polyethyleneimine [35]. Briefly, plasmids were diluted in DMEM, mixed with polyethyleneimine (PEI), and incubated for 15 min at room temperature. The mixture was then added to cells, followed by culture in DMEM supplemented with 10% FBS for 3 days.

### Tissue lysate Preparation

Mice were perfused with PBS, and tissues were dissected and snap-frozen with liquid nitrogen and kept at -80 °C. On the day of the experiment, frozen tissues were thawed and homogenized on ice with bead homogenizer (Moni International) in ice-cold RIPA buffer (150 mM NaCl, 50 mM Tris-HCl [pH 8.0], 1% Triton X-100, 0.5% sodium deoxycholate, 0.1% SDS) with 1 mM PMSF, and 1x protease inhibitors (Roche). After centrifugation at 14,000 × g for 15 min at 4℃, supernatants were collected for analysis. The insoluble pellets were washed with RIPA buffer and extracted in 2× v/w of Urea buffer (7 M Urea, 2 M Thiourea, 4% CHAPS, 30 mM Tris, pH 8.5). After sonication, samples were centrifuged at 200,000 g at 24 °C for 1 h, and the supernatant was collected as the Urea-soluble fraction.

### Protein purification

His-PGRN and sPLA2-IIA proteins were purified from the serum-free culture medium of transfected HEK293T cells using cobalt beads. Protein was eluted with imidazole. Purified proteins were further concentrated and changed to PBS buffer using the Centricon device (Millipore).

### SILAC proteomics analysis

HEPG2 cells were grown for a minimum of five generations in DMEM with 10% dialyzed FBS (Sigma) supplemented with either 12C14N or 13C15N Arginine and Lysine. To search for PGRN interactors, the heavy HEPG2 cells were transfected in two 15 cm dishes with GFP-PGRN expression constructs, while the light HEPG2 cells were transfected with pEGFP-C1 as a control. Two days after transfection, cells were lysed in 50mM Tris pH8.0, 150 mM NaCl, 1% Triton, and 0.1% deoxycholic acid with protease inhibitors (Roche). The lysates were subjected to anti-GFP immunoprecipitation using GFP-Trap beads (ChromoTek). The presence of GFP and GFP-PGRN in immunoprecipitated samples was confirmed by SDS-PAGE and silver staining. Samples were then mixed and boiled for 5 min with 1% DTT followed by alkylation by treating samples with a final concentration of 28 mM iodoacetamide. Proteins were precipitated on ice for 30 min with a mixture of 50% acetone/49.9% ethanol/0.1% acetic acid. The protein pellet was washed with the same solution and resolubilized in 8 M urea/50 mM Tris pH 8.0 followed by dilution with three volumes of 50 mM Tris pH 8.0/150 mM NaCl. Proteins were digested overnight on a nutator at 37ºC with 1 µg mass spectrometry grade Trypsin Gold (Promega). The resulting peptide sample was acidified at a final concentration of 0.25% formic acid and 0.25% trifluoroacetic acid (TFA) and desalted using a 50 mg Sep-Pak C18 cartridge (Waters). Samples were eluted with 80% acetonitrile (ACN)/0.1% acetic acid into silanized vials (National Scientific) and evaporated using a SpeedVac. Samples were reconstituted in 85 µL of 700:300:1 ACN: Water: FA and fractionated via hydrophilic interaction liquid chromatography (HILIC) on an Ultimate 300 LC (Dionex) using a TSKGel Amide-80 column (Tosoh bioscience). Each fraction was evaporated with a SpeedVac and resuspended in 0.1% TFA. Samples were subjected to LC-MS/MS analysis using a Q-Exactive HF mass spectrometer (Thermo Fisher Scientific), and data were analyzed using the SORCERER 2 system (Sage-N Research).

### Immunoprecipitation and Western blot analysis

Cells were lysed in 50 mM Tris pH 8.0, 150 mM NaCl, 1% Triton, and 0.1% deoxycholic acid with protease inhibitors (Roche). The lysates were subject to anti-GFP immunoprecipitation using GFP-Trap beads or anti-Myc immunoprecipitation using Myc-Trap beads (ChromoTek). The beads were washed with 50 mM Tris pH 8.0, 150 mM NaCl, 1% Triton after 3–4 h of incubation. Western Blot was visualized using the Licor-Odyssey system as described [36].

### Immunofluorescence staining and image acquisition

For mouse brain section staining, mice were perfused with cold PBS, and tissues were post-fixed with 4% paraformaldehyde. After dehydration in 30% sucrose buffer, tissues were embedded in the O.C.T compound (Electron Microscopy Sciences). 18-µm-thick brain sections were cut with a cryotome. Antigen retrieval was performed by microwaving in citrate buffer (pH 6.0) for 20 min. Tissue sections were blocked and permeabilized with 0.1% saponin in Odyssey blocking buffer before incubating with primary antibodies overnight at 4℃. The next day, sections were washed 3 times with cold PBS, followed by incubation with secondary fluorescent antibodies and Hoechst at room temperature for two hours. The slides were then mounted using a mounting medium (Vector Laboratories). Images were acquired on a CSU-X spinning disc confocal microscope (Intelligent Imaging Innovations) with an HQ2 CCD camera (Photometrics) using 63x or 100x objectives, and ten to twenty different random images were captured. Lower magnification images were captured by 10x or 20x objectives on a Leica DMi8 inverted microscope, and three to five images were captured from each sample.

### sPLA2 activity assays

sPLA2 activities were analyzed using a mouse sPLA2 activity assay kit (Cayman Chemical) according to the manufacturer’s instructions. Samples were prepared in a 96-well plate and run at 414 nm every minute for 10 min. The activities of recombinant sPLA2-IIA protein (250 ng/ml) in the absence or presence of recombinant PGRN or BSA (1250 ng/ml or 2500 ng/ml) were determined.

### Lipid measurement

To measure prostaglandin and free fatty acids levels, lipids were extracted from 50 mg of the frozen cortices and 80 µL of serum using the Bligh-Dyer protocol [37]. Briefly, 450 µL methanol/chloroform (2:1, v/v) was added to pulverized frozen brain tissues or serum, and the samples were vortexed. 120 µL of water was then added to each tissue suspension, and the samples were vortexed for 2 min, followed by centrifugation at 13,000 rpm for 2 min. 150 µL chloroform and water were then added sequentially, and the samples were vortexed and centrifugated as above after adding each reagent. The lower organic layers were collected as lipid extracts. The lipid extracts were evaporated under a stream of nitrogen, dissolved in 100 µL chloroform/methanol/water (73:23:3, v/v), and then analyzed by mass spectrometry (6230 Time-of-Flight, Agilent) coupled to high-performance liquid chromatography (1260 Infinity, Agilent). Samples were separated using normal phase with a luna silica column (3 μm, 50 × 2 mm, Phenomenex) and a binary gradient elution system where solvent A was chloroform/methanol/ammonium hydroxide (85:15:0.5, v/v) and solvent B was chloroform: methanol: water: ammonium hydroxide (60:34:5:0.5, v/v) under the flow rate of 0.5 mL/min. The following gradient time program (time [%A/%B]) was used: 0 min (100/0)-0.5 min (100/0)-9 min (0/100)-12 min (0/100)-15 min (100/0)-18.24 min (100/0). Compounds were detected using a Dual Agilent Jet Stream Electrospray Ionization source operated in negative mode with the following parameters: gas temperature, 325 ºC; drying gas, 10 L/min; nebulizer, 20 psig; sheath gas temperature, 400ºC; sheath gas flow, 12 L/min; VCap, 4000 V; Nozzle Voltage, 2000 V; fragmentor, 175 V m/z; mass range, 100–1500; acquisition rate, 1000 ms/spectrum. Data were acquired and converted to.mzdata files by MassHunter Workstation Software (Version B.08.00, Agilent) for the subsequent analysis. Signals within the range of mz = 351.225 ± 0.05 were integrated to generate the extracted ion chromatograms, and chromatographic peaks were integrated using Python 3.10.4 available at http://www.python.org. Peaks were only selected when their intensity exceeded three times the baseline noise. Prostaglandins were quantified using a standard curve generated from serial dilutions of standard PGE2 (P1884, TCI), which is estimated to be the most abundant prostaglandin with a de-protonated mass of 351.225 in the serum sample. The final prostaglandin concentrations were standardized per milliliter of serum or per milligram of brain tissue. For C18:1, C18:2, and C18:3 fatty acids measurement, the total peak area identified for each lipid species was measured, and the relative changes were analyzed.

### RNA-seq analysis

Total RNA was extracted from cortex regions using Trizol (Thermo Scientific). RNA quality was checked using a nanodrop, gel electrophoresis, and an Agilent Fragment Analyzer. RNA-seq libraries were generated by the Cornell TREx Facility using the NEBNext Ultra II Directional RNA Library Prep Kit (New England Biolabs) using 700ng input total RNA per sample. At least 20 M reads (2 × 150 nt PE) were generated on a NovaSeq (Illumina). Reads were trimmed to remove low-quality and adaptor sequences with TrimGalore (a wrapper for cutadapt and fastQC), requiring a minimum trimmed length of 50 nt. Reads that passed quality control were aligned to the reference genome (Ensembl 98.38) [38] using STAR [39], using ‘--quantMode GeneCounts’ to output counts per gene. SARTools [40] and DESeq2 [41] were used to generate normalized counts and statistical analysis of differential gene expression. Genes with FDR control p-value ≤ 0.05 and absolute log2 fold change ≥ 0.5 were identified as differentially expressed genes (DEGs). All the genes detected in RNA-seq were listed in the Supplemental Dataset S2. Gene enrichment analysis using Hallmark and KEGG gene sets was performed using Gene Set Enrichment Analysis (GSEA) [42]. The gene sets associated with inflammation were obtained from the GSEA hallmark database. The lysosome gene set is obtained from the GSEA KEGG database.

### Brain proteomics analysis

Mouse brain proteomics analysis was conducted as described previously [43]. Briefly, mouse brain hemisphere tissue samples were lyophilized, weighed, homogenized in a methanol: water solvent mixture (5:2, v: v), and centrifuged to obtain a protein pellet. The protein pellet was dissolved in lysis buffer (8 M urea, 50 mM ammonium bicarbonate (AmBC), 150 mM NaCl), sonicated in ice-cold water, and clarified by centrifugation. Protein concentration was measured by a colorimetric DCA assay (Bio-Rad), and 40 µg of protein from each sample was used for protein digestion. Protein lysates were reduced, alkylated, diluted, and digested with trypsin/Lys-C (1:25, w: w) overnight, followed by quenching with TFA till pH < 2. Peptides were cleaned using a Waters HLB 96-well plate, dried, and reconstituted in 2% ACN with 0.1% FA for LC-MS analysis on a Dionex Ultimate 3000 RSLCnano system coupled with a Q Exactive HF-X Orbitrap MS. Peptides were separated using an Easy-Spray PepMap RSLC C18 column (2 μm, 100 Å, 75 μm × 50 cm) with a 210-minute gradient at 55 °C and 0.25 µL/min flow rate. Data analysis was performed with Spectronaut software (v19.2, Biognosys) in DirectDIA mode, using the UniProt *Mus musculus* database alongside a custom contaminant library [43]. Carbamidomethylation was set as the fixed modification. Methionine oxidation and N-terminal acetylation were included as variable modifications. A false discovery rate (FDR) cutoff of 0.01 was applied to peptide and protein identifications. Precursor intensities below 1,000 were excluded from downstream analysis. Identified proteins were listed in Supplemental Dataset S3. Statistical significance was determined using an unpaired Student’s t-test, with proteins exhibiting FDR < 0.05 and absolute log_2_(FC) > 0.5 considered significantly altered. Pathway enrichment analysis was conducted via Metascape [44].

### Image analysis

For the quantitative analysis of GFAP, CD68, and IBA1 levels in the brain sections, the fluorescence intensity was measured directly using ImageJ after a threshold application. To quantify the CD68 levels in microglia, IBA1-positive cells were selected, and CD68 signals within microglia were measured using the region of interest (ROI) tool in ImageJ. The lipofuscin quantification was determined by the red fluorescence signals (594 nm). For the quantitative analysis of CathD and LAMP1 levels in microglia and neurons, the IBA1 or NeuN-positive cells were selected, and CathD and LAMP1 within these cells were measured using the ImageJ ROI tool. Three brain sections per mouse, separated by 100 μm, were used for quantification. The mean from the three sections was used to be representative of each mouse. Data from ≥ 3 brains in each genotype were used for quantitative analysis and normalized to age-matched controls.

### Statistical analysis

Statistical analyses were performed using GraphPad Prism 10. All data are presented as mean ± SEM. Statistical significance was assessed by unpaired Student’s t-test (for two-group comparison) or one-way ANOVA tests with Bonferroni’s multiple comparisons (for multiple comparisons). *P* values less than or equal to 0.05 were considered statistically significant. *p < 0.05; **p < 0.01; ***p < 0.001.

## Results

### Loss of PGRN in the FVB/N strain background leads to earlier gliosis and lysosomal abnormalities compared to the C57BL/6 background

**Fig. 1:**
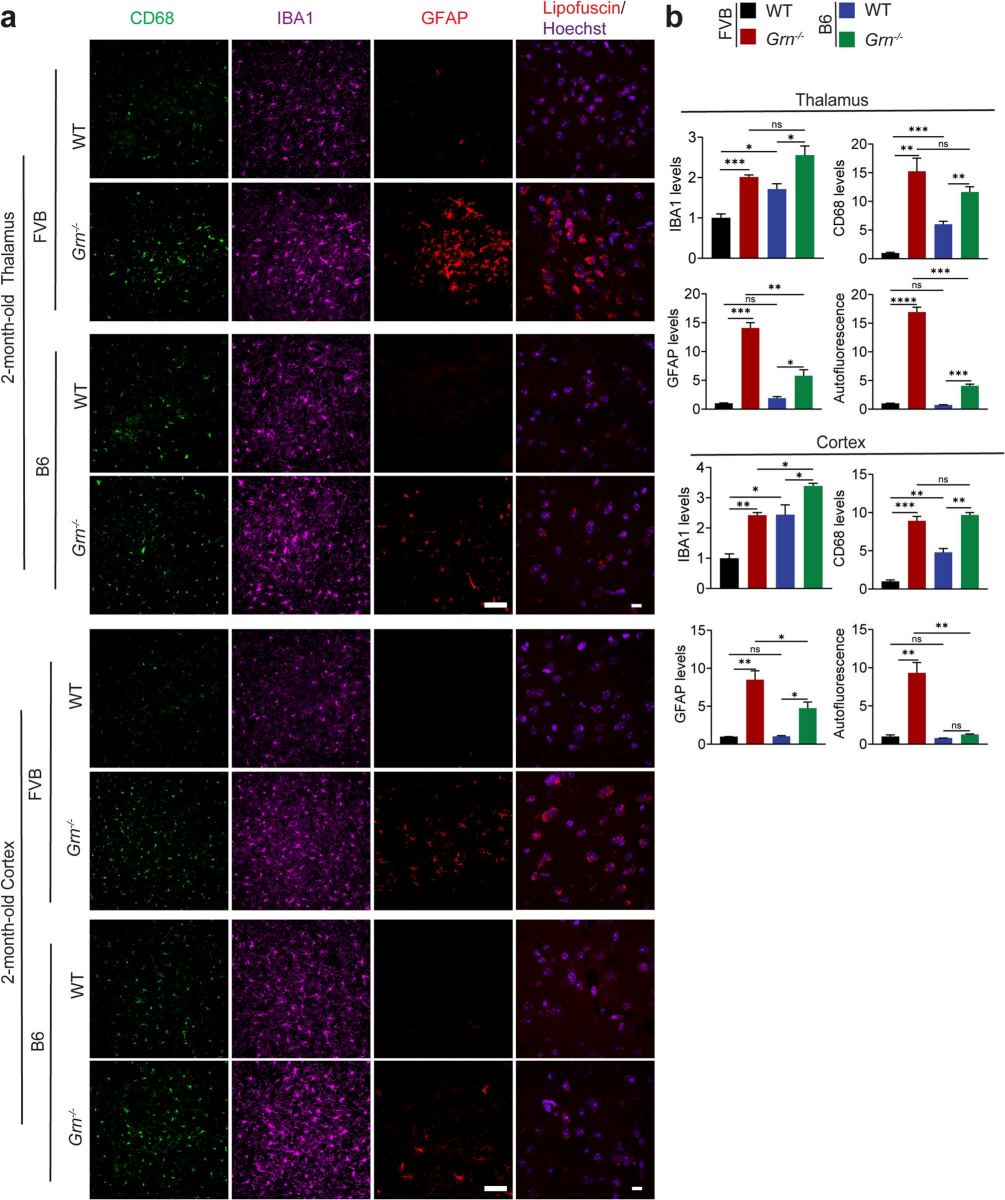
Glial activation and lipofuscin accumulation in 2-month-old PGRN-deficient FVB and B6 mice. (**a**) Immunostaining of GFAP, CD68, and IBA1 and auto-fluorescent lipofuscin signals in brain sections from 2-month-old WT and *Grn*^*−/−*^ mice in the FVB or B6 strain backgrounds. Representative images from the cortex and the thalamus were shown for GFAP/CD68/IBA1 staining and lipofuscin signals. Scale bar = 100 μm (GFAP/CD68/IBA1) and scale bar = 10 μm (lipofuscin). (**b**) Quantification of GFAP, CD68, and IBA1 levels and auto-fluorescent lipofuscin signals for the experiment in (a). Data are presented as mean ± SEM from 3 mice per group (*n* = 3). 3 brain sections were analyzed for each mouse brain. *, *p* < 0.05, **, *p* < 0.01, ***, *p* < 0.001, ****, *p* < 0.0001, Student’s t-test

To explore the effect of strain background on PGRN-deficient phenotypes, we crossed *Grn*^*−/−*^ mice in the B6 background with wild-type (WT) FVB mice. Brain pathology was examined in *Grn*^*−/−*^ mice in the FVB/B6 hybrid background to determine the consequences of PGRN loss. Interestingly, we found a significant increase in glial activation, lipofuscin accumulation, and enlarged lysosomes in neurons and microglia in the 2-month-old *Grn*^*−/−*^ mice (Fig. S1). To further confirm this phenotype, we backcrossed *Grn*^*−/−*^ mice into the FVB background for 6 additional generations and compared lipofuscin signals and glial activation in the 2-month-old *Grn*^*−/−*^ mice with littermate WT controls. Similar increases in glia activation and lipofuscin accumulation were observed (Fig. 1a and b), and most of the lipofuscin signals are found in NeuN + neurons and IBA1 + microglia but not in the GFAP + astrocytes (Fig. S2). In contrast, PGRN loss only leads to a subtle increase in gliosis and lipofuscin accumulation in the 2-month-old B6 mice (Fig. 1a and b).

**Fig. 2:**
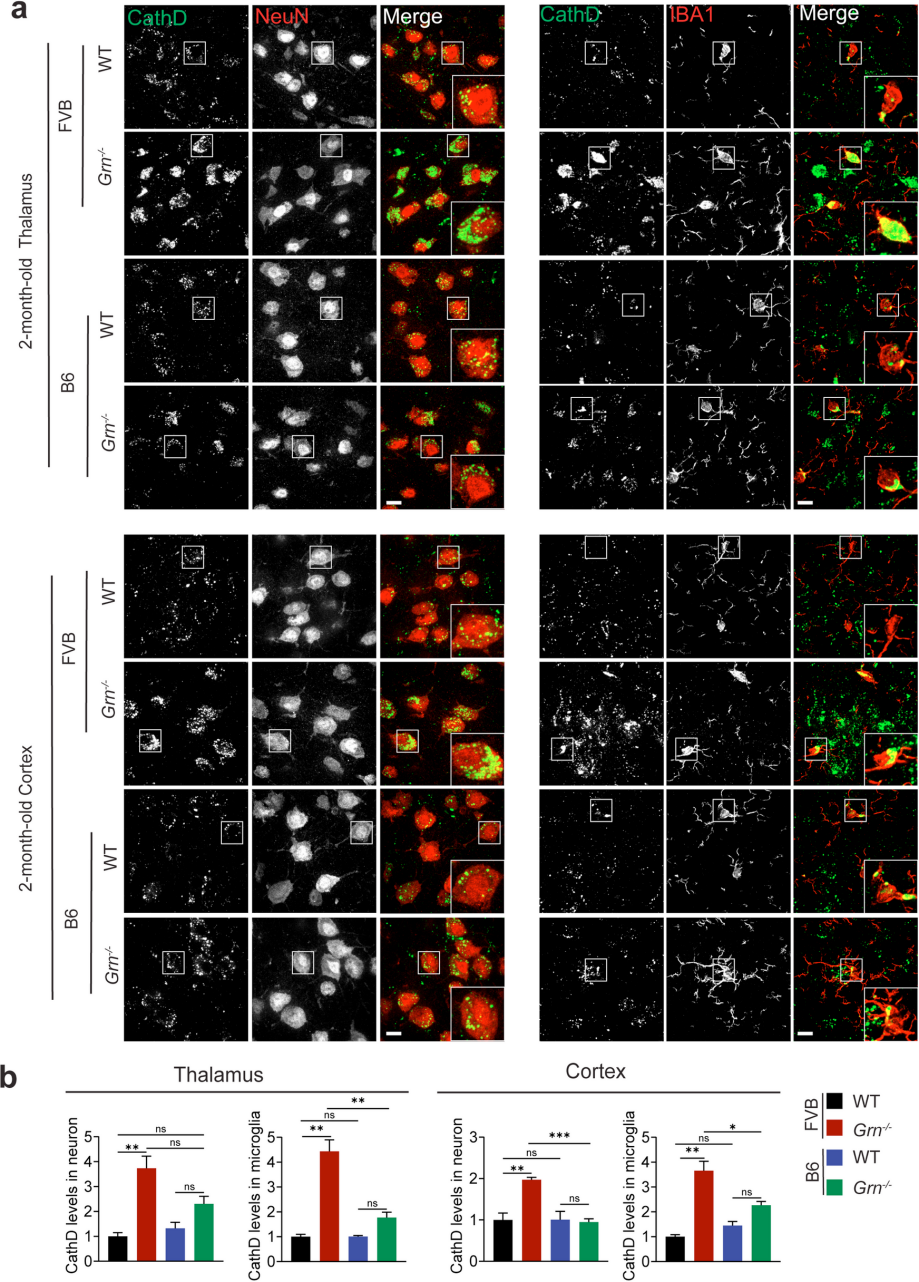
Lysosomal changes in 2-month-old PGRN-deficient FVB and B6 mice. (**a**,** b**) Immunostaining of Cathepsin D (CathD), NeuN, and IBA1 in brain sections from 2-month-old WT and *Grn*^*−/−*^ mice in FVB and B6 strain background. Representative images from the cortex and thalamus were shown. CathD signals in NeuN-positive neurons and IBA1-positive microglia were quantified (b). 3 sections per mouse brain and 3 mouse brains were used for each genotype. 15–20 microglia and 80–100 neurons per section were analyzed. Data are presented as mean ± SEM from 3 mice per group. *n* = 3, *, *p* < 0.05, **, *p* < 0.01, ***, *p* < 0.001. Student’s t-test. Scale bar = 10 μm

To further compare lysosomal phenotypes caused by PGRN deficiency in FVB versus B6 mouse strains, we immunostained 2-month-old WT and *Grn*^*−/−*^ mice with antibodies against lysosomal protein Cathepsin D, together with microglial marker IBA1. A significant increase in Cathepsin D levels was detected in PGRN-deficient microglia and neurons (Fig. 2a and b). In addition, enlarged lysosomes are frequently found in PGRN-deficient microglia and neurons (Fig. 2a and b), indicating early lysosomal defects in both microglia and neurons upon PGRN loss in the FVB mice. In contrast, PGRN deficiency in the B6 background only results in a subtle increase in Cathepsin D levels in neurons and microglia in the 2-month-old mice (Fig. 2a and b). In addition, because PGRN haploinsufficiency is a leading cause of FTLD, we examined the phenotype of the *Grn*^*+/−*^ mice in the FVB background. No obvious inflammation and lysosomal phenotypes were observed in these mice at 5 months old (Fig. S3), indicating that partial loss of PGRN is not sufficient to induce inflammatory and lysosomal abnormalities at this age, even in the FVB background. Taken together, PGRN deficiency causes an earlier onset of inflammation and lysosomal abnormalities in the FVB background compared to the B6 background.

### Loss of PGRN in the FVB/N mouse brain leads to exacerbated phenotypes during aging and increased levels of phosphorylated TDP-43

**Fig. 3:**
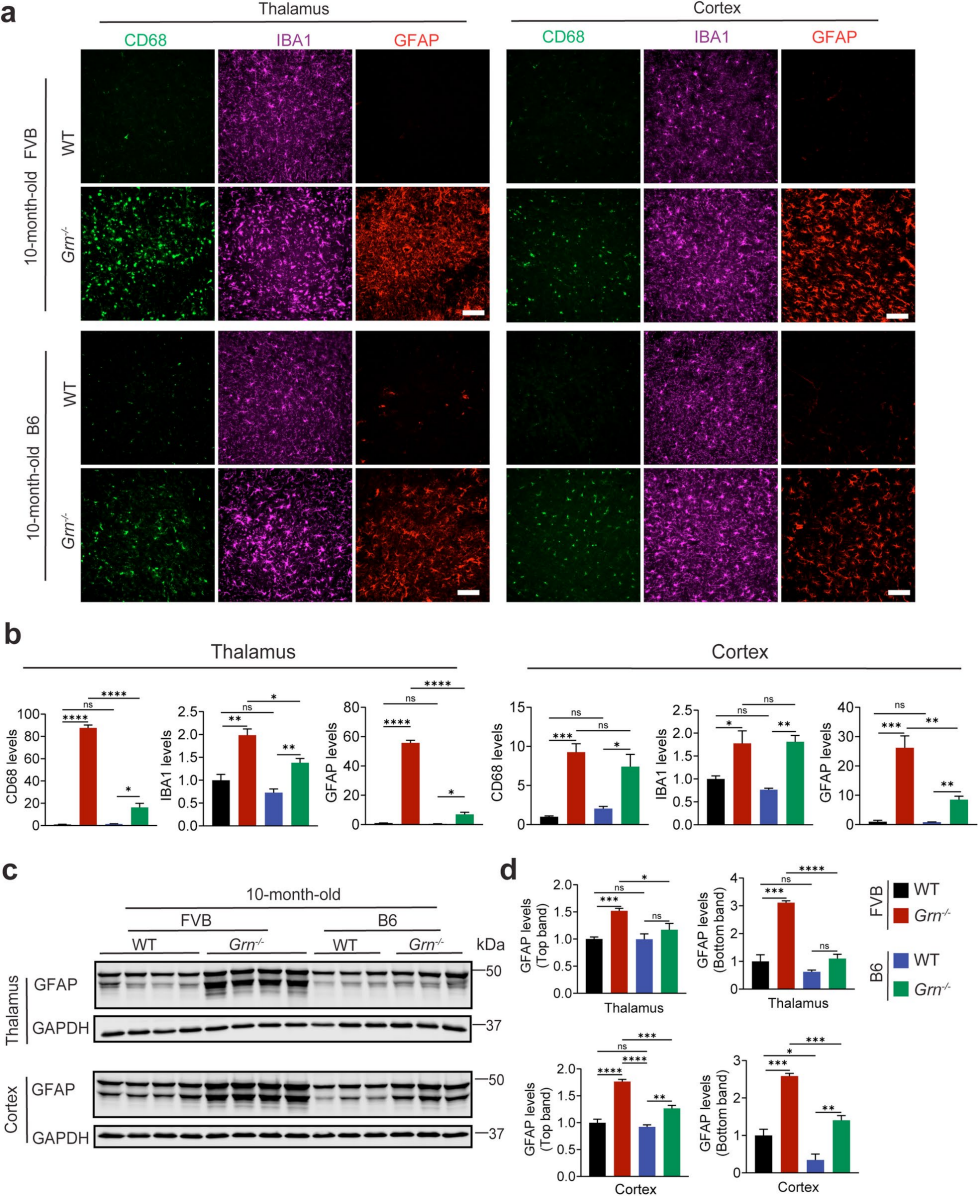
Glial activation in 10-month-old PGRN-deficient FVB and B6 mice. (**a**) Immunostaining of GFAP, CD68, and IBA1 in brain sections from 10-month-old WT and *Grn*^*−/−*^ mice in the FVB or B6 strain backgrounds. Representative images from the cortex and the thalamus were shown for GFAP/CD68/IBA1 staining (a). Scale bar = 100 μm. (**b**) Quantification of GFAP, CD68, and IBA1 levels in (a). Data are presented as mean SEM from 3–4 mice per group (*n* = 3–4). 3 brain sections were analyzed for each mouse brain. *, *p* < 0.05, **, *p* < 0.01, ***, *p* < 0.001, ****, *p* < 0.0001, Student’s t-test. (**c**,** d**) Western blot analysis of GFAP in the thalamus and cortex of WT and *Grn*^*−/−*^ mice in FVB and B6 backgrounds. The GFAP levels were quantified by using ImageJ and were normalized to the levels of the loading control GAPDH (D). Data are presented as mean ± SEM from 3–4 mice per group (*n* = 3–4). *, *p* < 0.05, ***, *p* < 0.001, ****, *p* < 0.0001. Student’s t-test

**Fig. 4:**
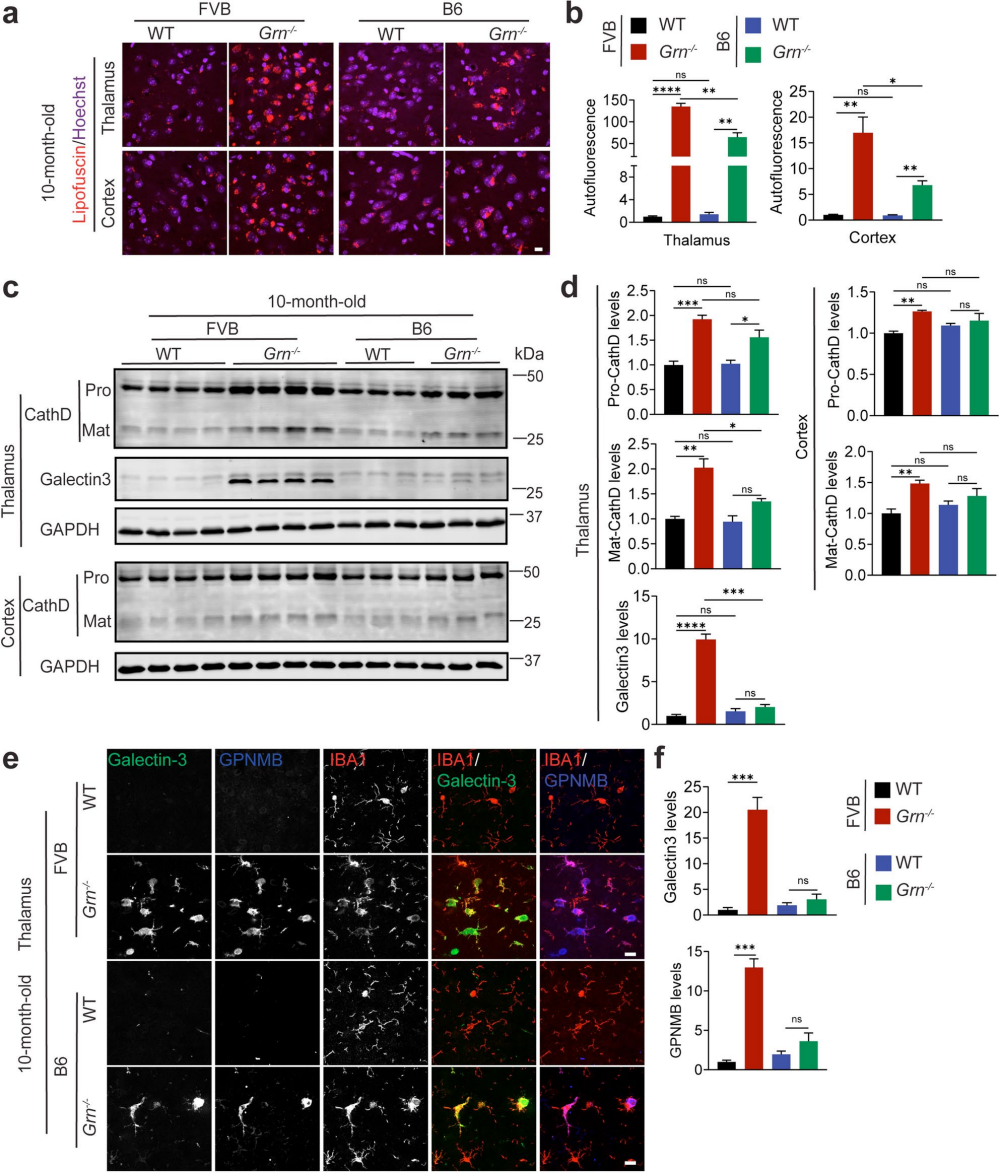
Lysosomal changes in 10-month-old PGRN-deficient FVB and B6 mice. (**a**,** b**) Lipofuscin accumulation in 10-month-old WT and *Grn*^*−/−*^ mice in FVB and B6 backgrounds. Representative images from the cortex and the thalamus were shown. Scale bar = 10 μm. Auto-fluorescent lipofuscin signals were quantified (B). (**c**,** d**) Western blot analysis of CathD and Galectin3 in the thalamus or cortex of WT and *Grn*^*−/−*^ mice in FVB and B6 backgrounds. The levels of both precursor (Pro-CathD) and mature form (Mat-CathD) of CathD, as well as Galectin3, were quantified by using ImageJ and were normalized to GAPDH levels (**d**). Data are presented as mean ± SEM from 3–4 mice per group (*n* = 3–4). **, *p* < 0.01, ***, *p* < 0.001, ****, *p* < 0.0001. Student’s t-test. (**e**,** f**) Immunostaining of Galectin-3, GPNMB, and IBA1 in brain sections from 10-month-old WT and *Grn*^*−/−*^ mice in the FVB and the B6 strain background. Representative images from the thalamus were shown. Galectin-3 and GPNMB levels in microglia in the thalamus were quantified in (**f**). Data are presented as mean ± SEM from 3–4 mice per group (*n* = 3–4), *, *p* < 0.05, **, *p* < 0.01, ***, *p* < 0.001. Student’s t-test. Scale bar = 10 μm

We further compared the PGRN deficiency phenotypes in 10-month-old FVB versus B6 mice. As expected, PGRN deficiency resulted in increased microglial and astrocyte activation as well as lipofuscin accumulation in both backgrounds (Figs. 3a and d and 4a and b). Apart from the comparable microglia activation in the cortex region between FVB and B6 *Grn*^*−/−*^ mice, changes in astrocyte activation and lipofuscin accumulation are more pronounced in both the thalamus and cortex of FVB *Grn*^*−/−*^ mice compared to B6 *Grn*^*−/−*^ mice, relative to WT (Figs. 3a and d and 4a and b). Additionally, levels of CathD in microglia and neurons in PGRN-deficient FVB and B6 mice were examined. CathD levels are significantly increased in the PGRN knockout mouse brain compared to WT in both backgrounds, with a further increase in FVB mice compared to the B6 mice in the thalamus but not in the cortex (Fig. 4c and d and Fig.S4a, 4b). Furthermore, we analyzed the levels of GPNMB and Galectin-3, two markers associated with microglial activation and lysosomal damage, which are known to be upregulated in PGRN-deficient microglia in mouse brains and FTLD-*GRN* brains [44–48]. These markers are increased in microglia in the thalamus of *Grn*^*−/−*^ mice in both backgrounds, with much more significant increases observed in FVB mice (Fig. 4c and f). These results suggest that the FVB background exacerbates gliosis and lysosomal phenotypes observed under PGRN-deficient conditions, especially in the thalamus region.

**Fig. 5:**
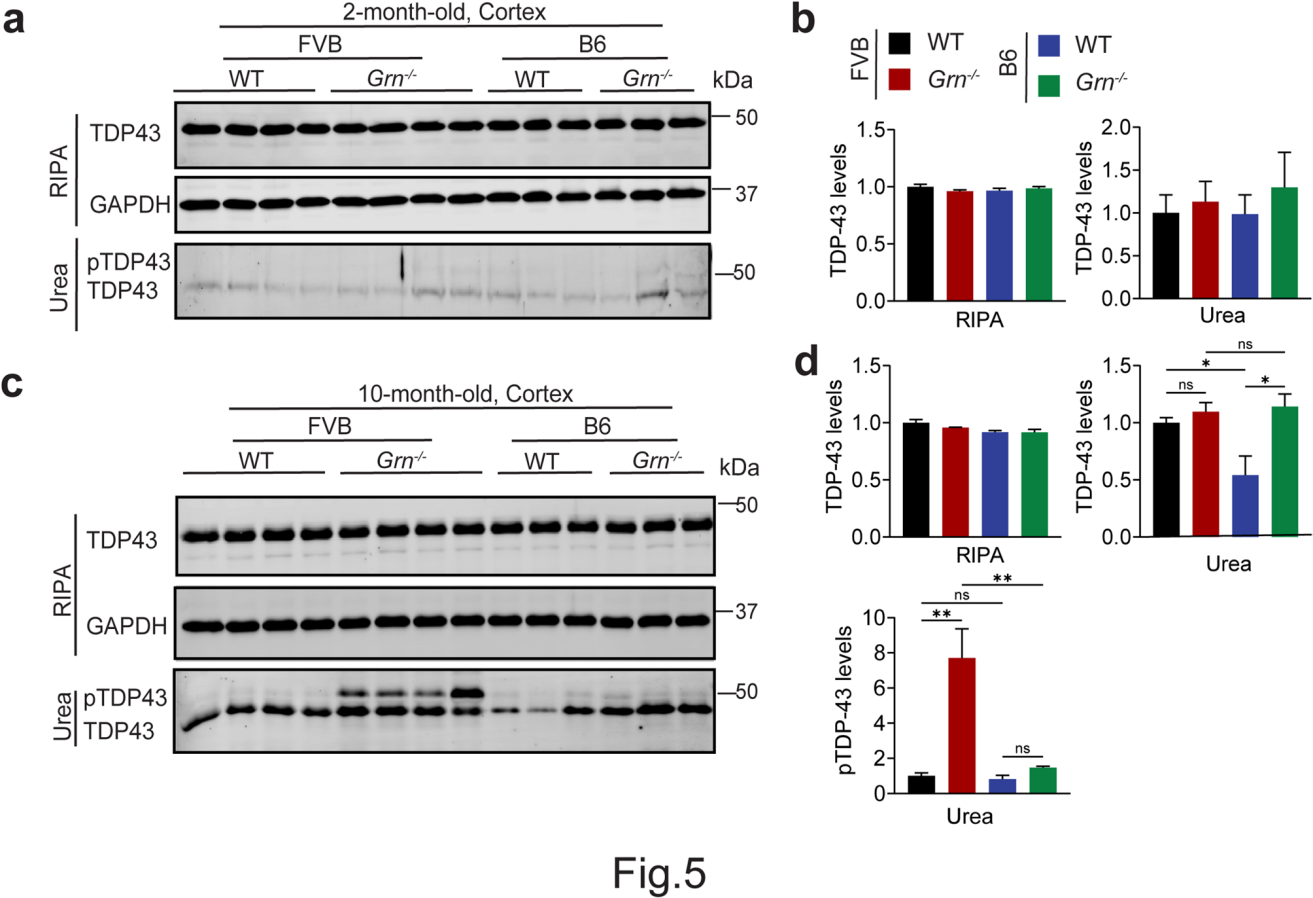
PGRN-deficiency in the FVB strain leads to enhanced TDP-43 pathology. (**a-d**) Analysis of TDP-43 solubility and phosphorylation in cortical lysates from 2- and 10-month-old WT and *Grn*^*−/−*^ mice in FVB and B6 strain backgrounds. Total TDP-43 levels and phosphorylated TDP-43 (pS409/410) levels in the RIPA-soluble and urea-soluble fractions of 2- and 10-month-old mice were quantified in (**b**) and (**d**), respectively. Data are presented as mean ± SEM from 3–4 mice per group (*n* = 3–4). *, *p* < 0.05, **, *p* < 0.01. Student’s t-test

TDP-43 aggregation is a hallmark of FTLD-*GRN*. We asked whether PGRN loss triggers TDP-43 pathology in FVB mice. Total and phosphorylation levels of TDP-43 in RIPA-soluble and urea-soluble fractions from 2- and 10-month-old *Grn*^*−/−*^ mice in both FVB and B6 backgrounds were analyzed. At 2 months old, PGRN deficiency barely affected the TDP-43 levels, solubility, and phosphorylation in both backgrounds (Fig. 5a and b). Interestingly, PGRN loss in the FVB background led to a significant increase in the levels of phosphorylated TDP-43 in urea-soluble fractions of 10-month-old mice (Fig. 5c and d), whereas PGRN loss in the B6 background had little effect (Fig. 5c and d), indicating that PGRN deficiency in the FVB background leads to enhanced TDP-43 pathology.

### PGRN physically interacts with sPLA2-IIA and regulates its activity in *vitro*

**Fig. 6:**
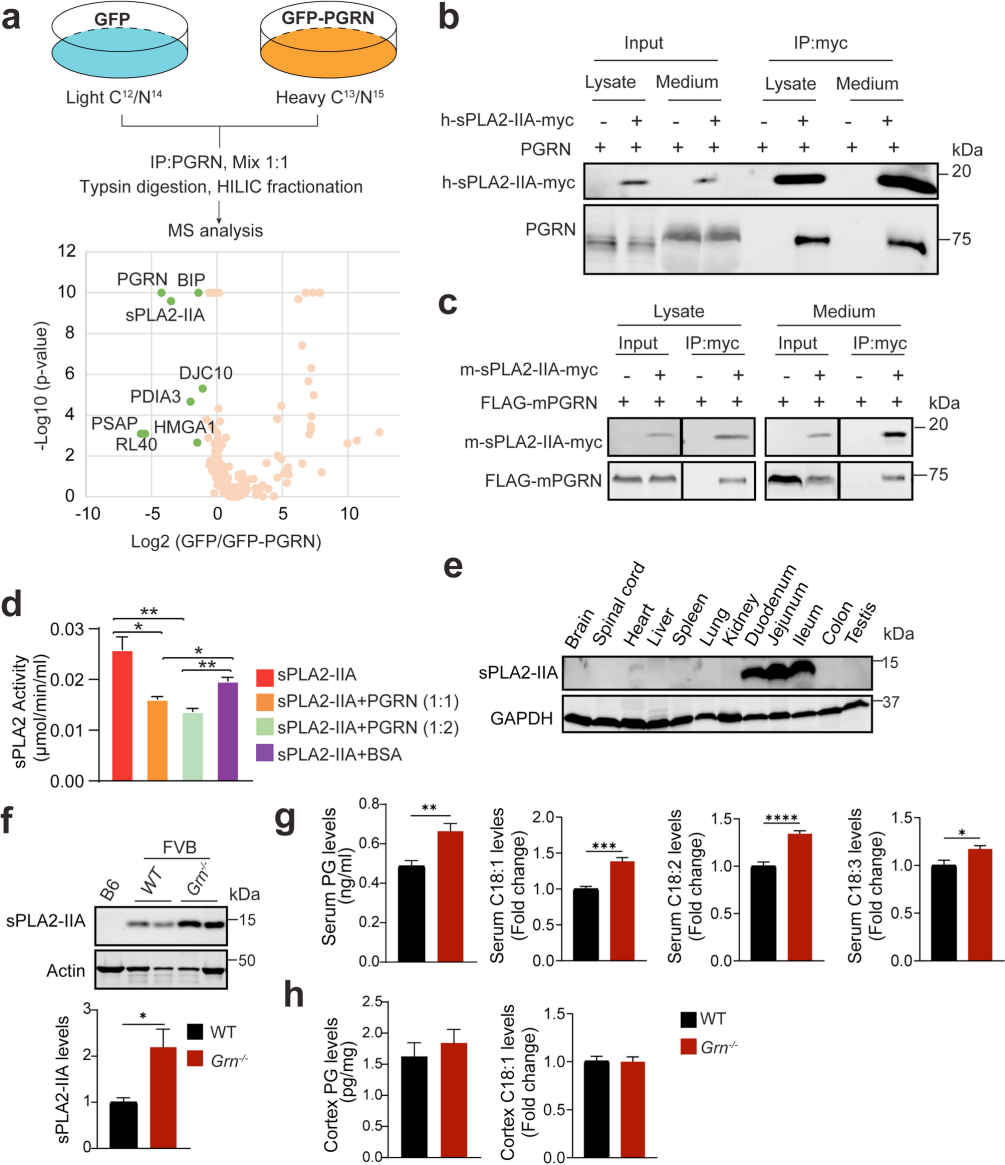
PGRN physically interacts with sPLA2-IIA and regulates sPLA2-IIA levels and activity. (**a**) Schematic illustration of the SILAC experiment searching for PGRN interactors in HEPG2 cells. Proteins with more than 4 peptides and p-value < 0.01 and Log2 (GFP/GFP-PGRN) ratio <-1 were labeled in green. (**b**) Human PGRN and sPLA2-IIA interact when overexpressed in HEK293T cells. Human sPLA2-IIA-myc and human PGRN-expressing constructs were transfected into HEK293T cells as indicated. Lysates and conditioned medium were prepared 3 days later and immunoprecipitated with anti-Myc antibodies. (**c**) Mouse-sPLA2-IIA-myc and FLAG-mouse-PGRN expressing constructs were transfected into HEK293T cells as indicated. Lysates and conditioned medium were prepared 3 days later and immunoprecipitated with anti-Myc antibodies. (**d**) PGRN inhibits sPLA2-IIA activities in vitro. Recombinant sPLA2-IIA protein was incubated with substrates in the absence or presence of recombinant PGRN at a 1:1 or 1:2 molar ratio, and sPLA2-IIA activities were measured. Data are presented as mean ± SEM from three independent experiments (*n* = 3). *, *p* < 0.05; **, *p* < 0.01, Student’s t-test. (**e**) The levels of sPLA2-IIA in tissue lysates from 2-month-old FVB mice were analyzed by western blot. (**f**) The levels of sPLA2-IIA are upregulated in the duodenum in PGRN-deficient mice. The duodenum of 2-month-old WT B6 mice and WT (*Pla2g2a*^*+/+*^*Grn*^*+/+*^) and *Grn*^*−/−*^ (*Pla2g2a*^*+/+*^*Grn*^*−/−*^) mice in the FVB background were lysed, and the same amount of the total protein was used for western blot to measure sPLA2-IIA levels. sPLA2-IIA levels in the mouse duodenum were quantified. Data are presented as mean ± SEM from 5 mice per group (*n* = 5). *, *p* < 0.05, Student’s t-test. (**g**,** h**) Serum levels of prostaglandin and C18:1, C18:2, and C18:3 fatty acids are upregulated in PGRN-deficient mice. Prostaglandin (predominantly PGE2 and PGD2, which have the same mass) concentration in serum and cortical lysates was measured by mass spectrometry using PGE2 as the standard. The levels of C18:1, C18:2, and C18:3 fatty acids were measured by mass spectrometry, and the relative abundance in WT and *Grn*^*−/−*^ mice was shown. Data are presented as mean ± SEM from six mice per group (*n* = 6). *, *p* < 0.05, **, *p* < 0.01, ***, *p* < 0.001, and ****, *p* < 0.0001, Student’s t-test

To understand the mechanisms of PGRN actions and dissect pathways affecting PGRN deficient phenotypes, we searched for PGRN interactors using a SILAC (stable isotope labeling by amino acids in cell culture) based proteomic screen (Fig. 6a). In addition to previously identified PGRN interactors, prosaposin (PSAP) [49] and folding chaperones (BIP, PDIA3, DJC10) [50], sPLA2-IIA (secreted phospholipase A2 type IIA, encoded by the *Pla2g2a* gene) was identified as one of the top hits from the screen (Fig. 6a, Supplementary Dataset 1). sPLA2-IIA is a secreted phospholipase of the sPLA2 family that catalyzes the hydrolysis of the sn-2 position of glycerophospholipids to generate fatty acids and lysophospholipids, many of which play a critical role in inflammation [51]. Interestingly, the B6 mouse strain harbors a naturally occurring mutation in *Pla2g2a*, which leads to the loss of the sPLA2-IIA protein in this background [52], while FVB mice express wild-type sPLA2-IIA. We hypothesized that sPLA2-IIA might explain the strain background difference observed for PGRN deficiency. To explore the relationship between PGRN and sPLA2-IIA, the PGRN-sPLA2-IIA interaction was first verified by co-immunoprecipitation (IP) with overexpressed human PGRN and sPLA2-IIA in both the medium and cell lysates (Fig. 6b). This interaction is conserved with mouse PGRN and mouse sPLA2-IIA (Fig. 6c). Next, we determined the functional significance of PGRN interaction with sPLA2-IIA. To test whether PGRN could directly regulate sPLA2-IIA activities, we purified recombinant sPLA2-IIA from the medium of transfected HEK293T cells and performed in *vitro* assays with colorimetric substrates of sPLA2 in the absence or presence of recombinant PGRN. We found that the presence of PGRN significantly reduced the activity of sPLA2-IIA at 1:1 and 1:2 molar ratios compared to the control reaction with sPLA2-IIA alone or with sPLA2-IIA and bovine serum albumin (BSA) at a 1:1 ratio (Fig. 6d).

### PGRN loss results in increased levels of sPLA2-IIA in the gut and elevated levels of prostaglandins and fatty acids in the serum

We next examined the effect of PGRN loss on sPLA2-IIA levels and functions in FVB mice. Consistent with previously published data [53], we found that the expression levels of sPLA2-IIA are not detectable in the brain but high in the intestine (Fig. 6e). Recently, sPLA_2_-IIA has been shown to regulate the gut microbiome, inflammation, and metabolism in the intestine [54–56]. Interestingly, we found that PGRN loss leads to a significant increase in sPLA2-IIA protein levels in the duodenum (Fig. 6f). Since sPLA2-IIA is a secreted enzyme, we next attempted to determine changes in the levels of secreted sPLA2-IIA. Unfortunately, we could not measure serum levels of sPLA2-IIA because we failed to identify a commercial ELISA kit specific to mouse sPLA2-IIA (data not shown). Thus, we instead decided to measure alterations in the levels of downstream products of sPLA2, prostaglandins, and free fatty acids [51, 57, 58]. We found a significant increase in the serum levels of prostaglandins PGE2 and PGD2, as well as C18:1, C18:2, and C18:3 fatty acids in PGRN-deficient mice (Fig. 6g). Interestingly, this increase is not found in the cortex lysates from PGRN-deficient mice (Fig. 6h).

### sPLA2 inhibitors ameliorate phenotypes associated with PGRN loss in the mouse brain

**Fig. 7:**
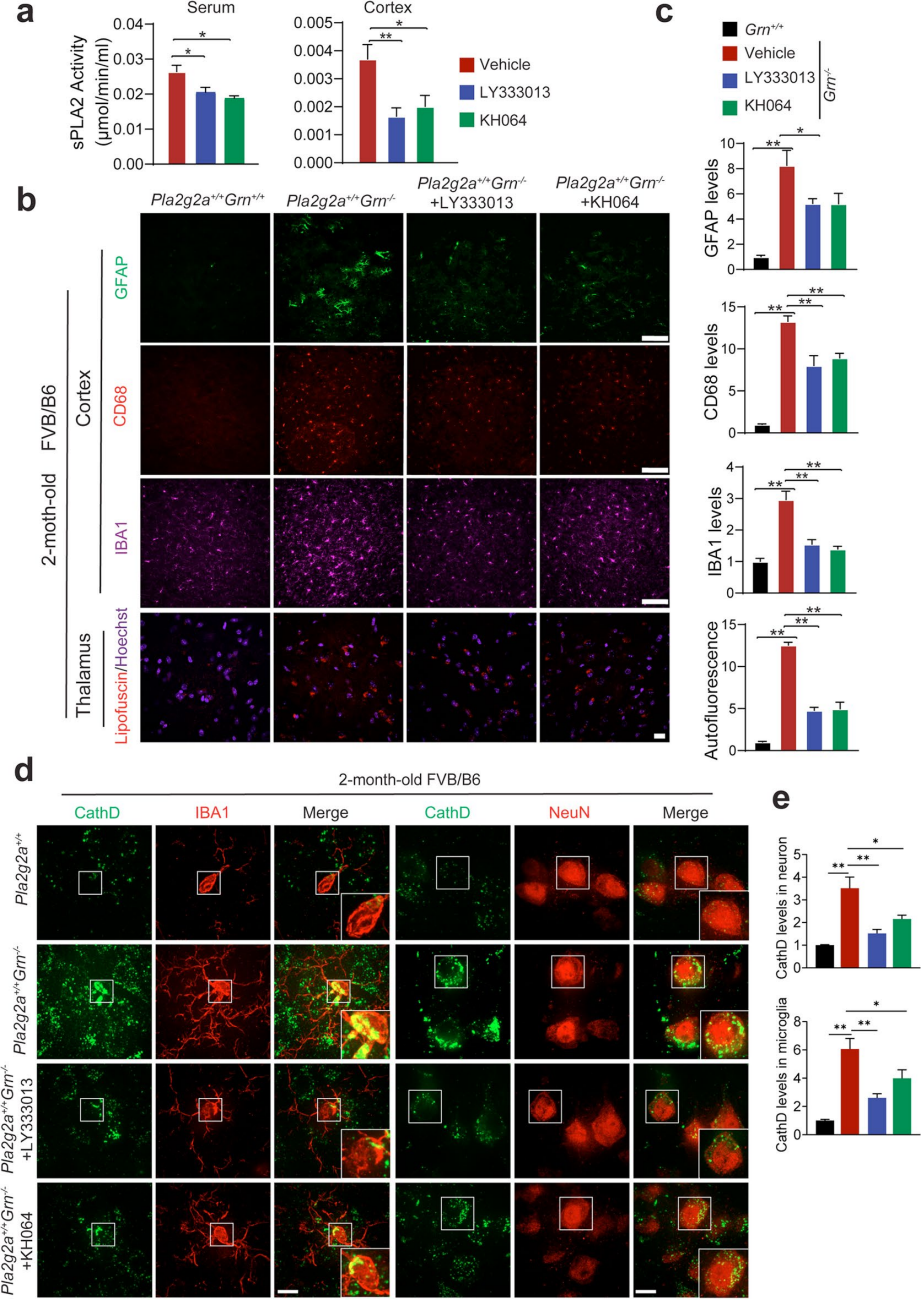
sPLA2 inhibitor treatment rescues PGRN deficiency phenotypes in mice. 5-week-old *Pla2g2a*^*+/+*^*Grn*^*+/+*^ and *Pla2g2a*^*+/+*^*Grn*^*−/−*^ mice in FVB/B6 mixed background were treated with corn oil control or sPLA2 inhibitors, LY333013 or KH064 for 3 weeks. (**a**) Measurement of total sPLA2 activity in the serum and cortical lysates from untreated and LY333013 or KH064-treated mice. Data are presented as mean ± SEM from five mice per group (*n* = 5). *, *p* < 0.05; **, *p* < 0.01, Student’s t-test. (**b**) Brain sections were immunostained with anti-GFAP, CD68, and IBA1 antibodies, and representative images from the cortex region were shown (Scale bar = 100 μm). Another set of brain sections was stained with Hoechst and auto-fluorescent lipofuscin signals (red) in the thalamus region were imaged (scale bar = 10 μm). (**c**) Quantification of GFAP, CD68, and IBA1 levels and auto-fluorescent lipofuscin signals for the experiment in (b). Data are presented as mean ± SEM from five mice per group (*n* = 5). 3 sections were analyzed for each mouse brain. *, *p* < 0.05; **, *p* < 0.01, Student’s t-test. (**d**) Brain sections were immunostained with anti-CathD, NeuN, and IBA1 antibodies, and representative images from the cortex region were shown (Scale bar = 10 μm). (**e**) CathD signals in IBA1-positive microglia and NeuN-positive neurons were quantified. 10–20 confocal images were randomly captured from each brain section. Ten to twenty microglia and fifty to sixty neurons were quantified per section. 3 sections were analyzed for each mouse brain. Data are presented as mean ± SEM from 3–4 mice per group (*n* = 3–4). *, *p* < 0.05; **, *p* < 0.01, Student’s t-test

Because sPLA2-IIA is an important mediator of inflammation through the generation of arachidonic acid (AA) and other fatty acid signaling molecules [51, 57, 58], we hypothesized that PGRN might regulate inflammation through sPLA2-IIA. To determine whether increased sPLA2-IIA activities contribute to the inflammation and lysosomal phenotypes in PGRN-deficient mice, we treated 5-week-old *Pla2g2a*^+/+^*Grn*^*−/−*^ mice in the mixed FVB/B6 background with LY333013, a broad inhibitor of the sPLA2 family phospholipases, or KH064, a relatively specific inhibitor of sPLA2-IIA [57]. Both inhibitors significantly decreased sPLA2 activities toward a commercial substrate in the serum and in the cortical lysates, indicating that the inhibitors have crossed the blood-brain barrier (Fig. 7a). Strikingly, the inhibition of sPLA2 not only rescues the microgliosis and astrogliosis phenotypes but also lipofuscin accumulation in *Grn*^*−/−*^ mice in the *Pla2g2a*^+/+^ background at 2-month-old (Fig. 7b and c). Enlarged lysosomes and increased levels of cathepsin D in neurons and microglia of *Pla2g2a*^+/+^*Grn*^*−/−*^ mice are also rescued by treatment with sPLA2s inhibitors (Fig. 7d and e). These results support that the increase in sPLA2-IIA activity might contribute to enhanced inflammation and lysosomal abnormalities in the *Pla2g2a*^+/+^*Grn*^*−/−*^ mice.

### AAV-mediated sPLA2-IIA overexpression leads to enhanced glial activation and lysosomal abnormalities in PGRN-deficient mice

**Fig. 8:**
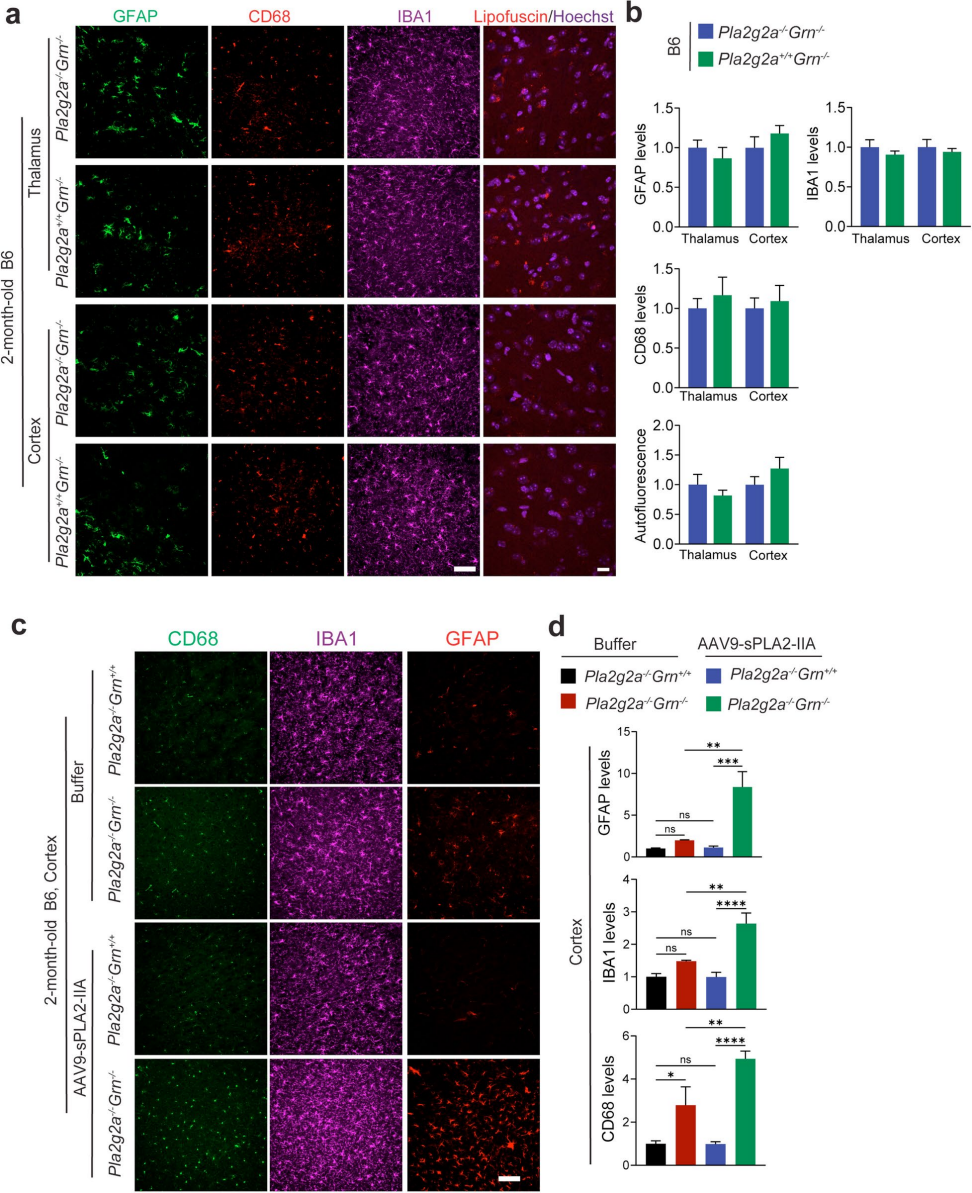
AAV9-mediated overexpression of sPLA2-IIA leads to enhanced glial activation in 2-month-old B6 *Grn*^*−/−*^ mice. (**a**,** b**) Expression of sPLA2-IIA at endogenous levels in *Grn*^*−/−*^ B6 mice does not affect glial activation and lipofuscin accumulation. Immunostaining of GFAP, CD68, and IBA1 and autofluorescent lipofuscin signals in brain sections from 2-month-old *Pla2g2a*^*−/−*^*Grn*^*−/−*^ and *Pla2g2a*^*+/+*^*Grn*^*−/−*^ littermate mice in the B6 background. Representative images from the cortex and the thalamus were shown for GFAP/CD68/IBA1 staining and lipofuscin signals. Scale bar = 100 μm (for GFAP/CD68/IBA1) and 10 μm (for lipofuscin). GFAP, CD68, and IBA1 levels and auto-fluorescent lipofuscin signals were quantified in (**b**). Data are presented as mean ± SEM from 4 mice per group (*n* = 4). 3 brain sections were analyzed for each mouse brain. (**c**,** d**) AAV9-mediated overexpression of sPLA2-IIA leads to enhanced glial activation in 2-month-old *Grn*^*−/−*^ mice in B6 mice. B6 WT (*Pla2g2*^*−/−*^*Grn*^*+/+*^) and *Grn*^*−/−*^ (*Pla2g2a*^*−/−*^*Grn*^*−/−*^) pups at postnatal day (P0) were injected with AAV9-sPLA2-IIA or the buffer control, and the mice were collected at 2 months old. (**c**) Brain sections were immunostained with anti-GFAP, CD68, and IBA1 antibodies, and representative images from the cortex and thalamus regions were shown (Scale bar = 100 μm). (**d**) Quantification of GFAP, CD68, and IBA1 levels for the experiment in (c). Data are presented as mean ± SEM from three to five mice per group (*n* = 3–5). Three sections were analyzed for each mouse brain. *, *p* < 0.05; **, *p* < 0.01; ****, *p* < 0.0001, Student’s t-test

**Fig. 9:**
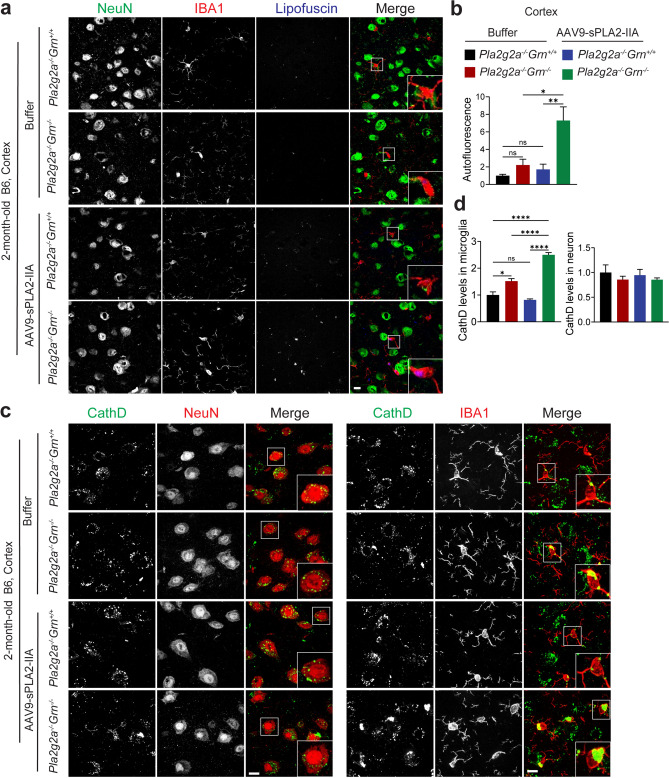
AAV9-mediated overexpression of sPLA2-IIA leads to lipofuscin accumulation and lysosomal enlargement in microglia in 2-month-old B6 *Grn*^*−/−*^ mice. B6 WT (*Pla2g2*^*−/−*^*Grn*^*+/+*^) and *Grn*^*−/−*^ (*Pla2g2a*^*−/−*^*Grn*^*−/−*^) pups at postnatal day (P0) were injected with AAV9-sPLA2-IIA or the buffer control, and the mice were collected at 2 months old. (**a**) Brain sections were stained with NeuN and IBA1, and auto-fluorescent lipofuscin signals (blue) in the cortex region were imaged (scale bar = 10 μm). (**b**) Quantification of autofluorescent lipofuscin signals in the cortex. Data are presented as mean ± SEM from three to five mice per group (*n* = 3–5). Three sections were analyzed for each mouse brain. *, *p* < 0.05; **, *p* < 0.01; ****, *p* < 0.0001, Student’s t-test. (**c**) Brain sections were immunostained with anti-CathD, NeuN, and IBA1 antibodies, and representative images from the cortex region were shown (Scale bar = 10 μm). (**d**) CathD signals in IBA1-positive microglia and NeuN-positive neurons were quantified. 10–20 confocal images were randomly captured from each brain section. Fifteen to thirty microglia and fifty to sixty neurons were quantified per Sect. 3 sections were analyzed for each mouse brain. Data are presented as mean ± SEM from 3–5 mice per group (*n* = 3–5). *, *p* < 0.05; **, *p* < 0.01; ****, *p* < 0.0001, Student’s t-test

Next, we test whether sPLA2-IIA expression is sufficient to drive inflammation and lysosomal abnormalities. We found that expression of either mouse or human sPLA2-IIA in WT B6 mice does not cause glial activation or lipofuscin accumulation (Fig.S5 and Fig.S6). We also generated *Pla2g2a*^+/+^*Grn*^*−/−*^ mice in the B6 background by crossing FVB *Grn*^*−/−*^ mice (*Pla2g2a*^*+/+*^*Grn*^*−/−*^) with B6 *Grn*^*−/−*^ mice (*Pla2g2a*^*−/−*^*Grn*^*−/−*^) and compared their phenotypes with those of *Pla2g2a*^−/−^*Grn*^*−/−*^ mice in the B6 background. We found that expression of sPLA2-IIA at endogenous levels does not enhance the glial activation and lipofuscin accumulation in 2-month-old *Grn*^*−/−*^ mice in the B6 background (Fig. 8a and b), indicating that basal levels of sPLA2-IIA are not sufficient to affect PGRN deficiency phenotypes. To further investigate the role of sPLA2-IIA in driving PGRN deficiency phenotypes, we overexpressed sPLA2-IIA in WT and *Grn*^*−/−*^ mice in the B6 strain background via AAV injection. Postnatal (P0) pups were injected with AAV9 viruses overexpressing sPLA2-IIA or buffer control orbitally. Mice were collected at 2 months old, and gliosis and lysosomal phenotypes were examined in brain sections. We found that sPLA2-IIA is highly expressed in the neurons in the cortex, but its expression in the thalamus region was minimal (Fig. S7a). An obvious colocalization between sPLA2-IIA and PGRN was observed (Fig. S7b), indicating that sPLA2-IIA is localized in the lysosomal compartment in neurons. Interestingly, we found that sPLA2-IIA overexpression in the cortex led to enhanced gliosis and lipofuscin accumulation in *Grn*^*−/−*^ mice but not in WT mice (Figs. 8c and d and 9a and b), suggesting that elevated sPLA2-IIA activity in the brain can only drive gliosis or lysosomal phenotypes when combined with PGRN loss. More intriguingly, the increased lipofuscin accumulation was found specifically in the microglia but not in neurons from the *Grn*^*−/−*^ mouse brain cortex region (Fig. 9a and b). Consistently, the levels of lysosomal enzyme CathD were significantly increased in microglia in PGRN-deficient mice upon sPLA2-IIA overexpression (Fig. 9c and d). These results further demonstrate that elevated sPLA2-IIA activities in PGRN-deficient mice enhance inflammation and lysosomal abnormalities.

### PGRN deficiency in FVB/N and C57BL/6 mouse strains leads to distinct alterations in transcriptome and proteome

**Fig. 10:**
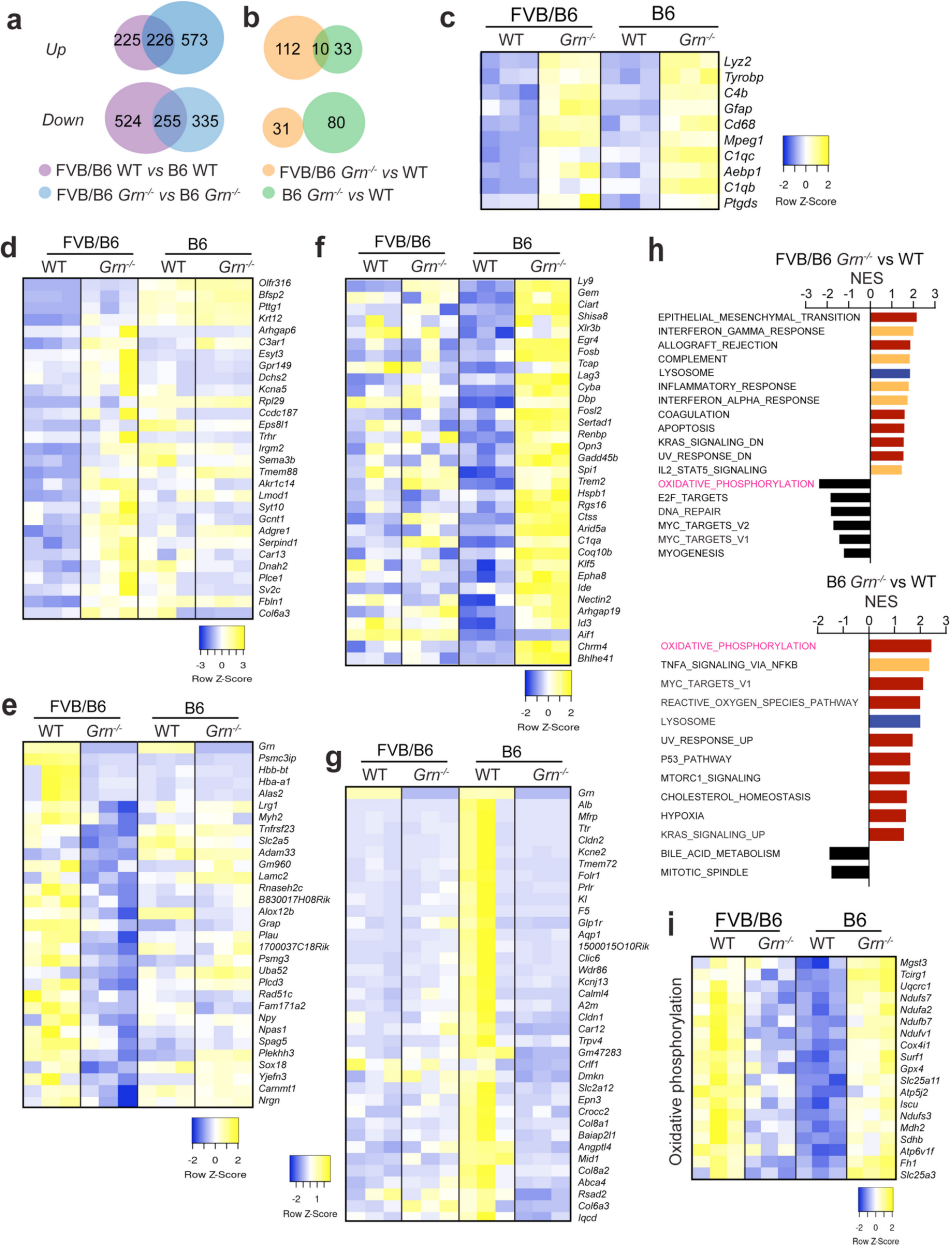
Gene expression changes caused by PGRN deficiency in the FVB/B6 and B6 mouse strains. Total RNAs were extracted from the cortex of 5-month-old WT and *Grn*^*−/−*^ mice in B6 and FVB/B6 mixed background, and the RNA-seq was performed to analyze gene expression changes (*n* = 3). (**a**,** b**) Venn diagram showing the overlap of the differentially expressed genes (DEGs) (FDR ≤ 0.05,|Log2FC| ≥ 0.5) identified in the indicated comparisons. (**c**) Heatmap showing the expression changes of genes significantly upregulated in *Grn*^*−/−*^ mice compared to WT in both B6 and FVB/B6 mixed backgrounds. DEGs with FDR ≤ 0.05 and log2(FC) ≥ 0.5 were plotted. (**d**) Heatmap showing the expression changes of genes specifically upregulated in *Grn*^*−/−*^ mice compared to WT in the FVB/B6 mixed background. DEGs with FDR ≤ 0.05 and log2(FC) ≥ 1.0 were plotted. (**e**) Heatmap showing the expression changes of genes specifically downregulated in *Grn*^*−/−*^ mice compared to WT in FVB/B6, the mixed background. DEGs with FDR ≤ 0.05 and log2(FC) ≤ -0.5 were plotted. (**f**) Heatmap showing the expression changes of genes specifically upregulated in *Grn*^*−/−*^ mice compared to WT in the B6 background. DEGs with FDR ≤ 0.05 and log2(FC) ≥ 0.5 were plotted. (**g**) Heatmap showing the expression changes of genes specifically downregulated in *Grn*^*−/−*^ mice compared to WT in the B6 background. DEGs with FDR ≤ 0.05 and log2(FC) ≤ -1.0 were plotted. (**h**) GSEA pathway enrichment analysis to identify the significantly upregulated and downregulated gene sets (FDR q-value < 0.1) in the indicated comparisons. The gene sets with normalized enrichment score (NES) >1.0 and those with NES < -1 are listed as upregulated or downregulated in the indicated groups. The gene sets associated with inflammation are highlighted in orange, and the lysosome gene set is highlighted in blue. (**i**) Heatmap showing the expression changes of the genes involved in oxidative phosphorylation

Since endogenous sPLA2-IIA expression in the B6 background is not sufficient to cause PGRN deficiency phenotypes, we performed an RNA-seq analysis with RNAs isolated from cortical regions of 5-month-old WT and *Grn*^*−/−*^ mice in a B6 versus FVB/B6 mixed background to gain more insight into the mechanisms driving the phenotypic differences caused by PGRN loss in FVB and B6 backgrounds. These analyses revealed substantial differences in gene expression between the two strain backgrounds (Fig. 10a and Supplementary Dataset 2). Additionally, only 10 genes overlap in the lists of differentially expressed genes (DEGs) identified in *Grn*^*−/−*^ mice compared to WT in the two strain backgrounds (Fig. 10b and c). These genes upregulated in *Grn*^*−/−*^ mice are all related to immune functions (Fig. 10c). Other genes that are specifically impacted by PGRN deficiency in each strain background are depicted in the heatmaps (Fig. 10d and g and Supplementary Dataset 2). Pathway analysis further revealed that different pathways are enriched in PGRN-deficient mice across two strain backgrounds (Fig. 10h). Nevertheless, lysosome and inflammation pathways are among the most upregulated pathways in both strain backgrounds upon PGRN loss (Fig. 10h), consistent with the known roles of PGRN. Notably, the oxidative phosphorylation pathway is significantly downregulated under PGRN deficiency in the FVB/B6 mixed background, whereas it is upregulated in the B6 background (Fig. 10h and i), suggesting that it might contribute to the phenotypic differences caused by PGRN loss in these two strain backgrounds.

**Fig. 11:**
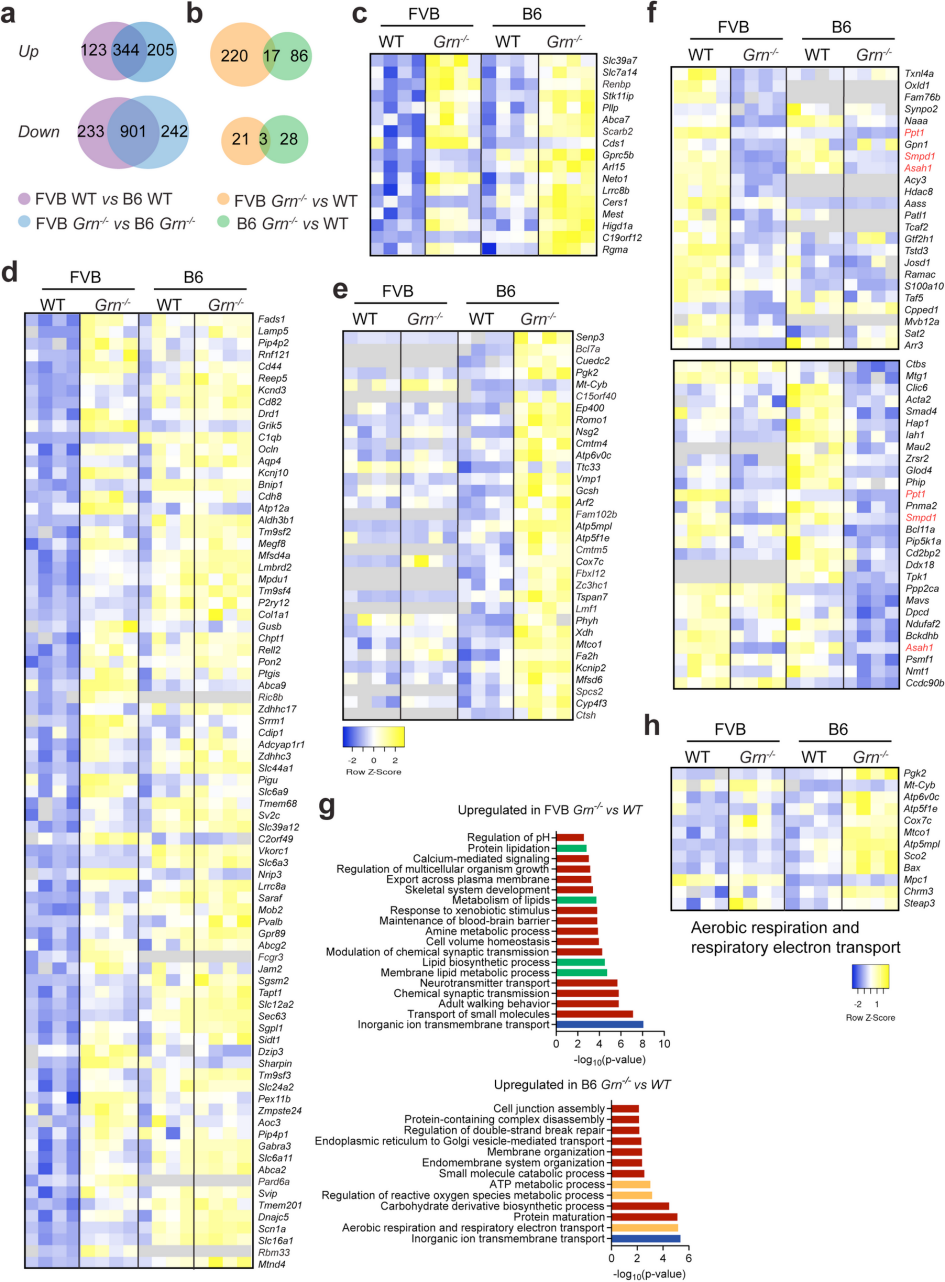
Proteomic changes caused by PGRN deficiency in the FVB and B6 mouse strains. Total proteins were extracted from brain hemispheres of 2-month-old WT and *Grn*^*−/−*^ mice in FVB and B6 backgrounds, and the proteomic analysis was performed to analyze protein changes (*n* = 4). (**a**,** b**) Venn diagram showing the overlap of the significantly changed proteins (FDR ≤ 0.05,|log2FC| ≥0.5) identified in the indicated comparisons. (**c**) Heatmap showing the expression of the proteins that were significantly upregulated (FDR ≤ 0.05, log2FC ≥ 0.5) in *Grn*^*−/−*^ mice compared to WT in both FVB and B6 backgrounds. (**d**) Heatmap showing the expression of the proteins (FDR ≤ 0.05, log2FC ≥ 1) specifically upregulated in FVB *Grn*^*−/−*^ compared with WT. (**e**) Heatmap showing the expression of the proteins (FDR ≤ 0.05, log2FC ≥ 1) specifically upregulated in B6 *Grn*^*−/−*^ mice compared with WT. (**f**) Heatmap showing the expression of the significantly downregulated proteins identified in FVB *Grn*^*−/−*^ mice compared with WT (top) and the downregulated proteins identified in B6 *Grn*^*−/−*^ mice compared with WT (bottom) (FDR ≤ 0.05 and log2FC ≤ -0.5). The proteins downregulated in *Grn*^−/−^ mice in both backgrounds are highlighted in red. (**g**) Upregulated pathways in *Grn*^*−/−*^ mice compared to WT mice in FVB (top) or B6 (bottom) background. Pathway enrichment analysis using gene ontology biology process (GOBP) and Reactome gene sets was performed using Metascape. The enriched pathways with *p*-value <0.01 were shown. The pathways associated with lipid metabolism, mitochondrial function, and inorganic ion transmembrane transport are highlighted in green, orange, and blue, respectively. (**h**) Heatmap showing the changes in the proteins involved in aerobic respiration and the aerobic respiratory electron pathway. Only those proteins with an intensity above 1,000 are displayed in the heatmap

We also performed proteomic analyses to identify protein changes triggered by PGRN loss in FVB and B6 backgrounds. Proteomic comparison of proteins in the brain lysates of 2-month-old FVB versus B6 mice identified approximately 1,600 DEGs (Fig. 11a and Supplementary Dataset 3). PGRN loss leads to more protein changes in the FVB mice compared to B6, as expected (Fig. 11b and Supplementary Dataset 3). However, minimal overlap between protein changes is observed in the two strain backgrounds upon PGRN loss (Fig. 11b). Seventeen proteins are significantly increased in *Grn*^*−/−*^ mice in both backgrounds (Fig. 11b and c), such as solute carrier family members SLC39A7 and SLC7A14, which function as zinc and amino acid transporters, respectively. Three proteins involved in lipid metabolism, including palmitoyl-protein thioesterase 1 (PPT1), sphingomyelin phosphodiesterase 1 (SMPD1), and N-acylsphingosine amidohydrolase 1 (ASAH1), were decreased in both FVB and B6 *Grn*^*−/−*^ mice compared to WT (Fig. 11f). Other proteins specifically affected by PGRN deficiency in each strain background are displayed in the heatmaps (Fig. 11d and e). Furthermore, pathway analysis revealed that inorganic ion transmembrane transport is the only pathway upregulated by PGRN loss in both backgrounds (Fig. 11g). Interestingly, multiple lipid metabolism pathways are significantly upregulated in *Grn*^*−/−*^ mice in the FVB but not B6 background, while pathways associated with mitochondrial function, such as aerobic respiration and respiratory electron transport, ATP metabolic process, and regulation of reactive oxygen species metabolic process, were specifically upregulated by PGRN deficiency in B6 but not FVB background (Fig. 11g and h). These findings suggest that alterations in lipid metabolism and mitochondrial function might be associated with the phenotypic differences caused by PGRN loss in the two strain backgrounds.

Taken together, the above results suggest that PGRN loss causes substantial differences in alterations in transcriptome and proteome in the FVB and B6 strain background, which could potentially modify PGRN deficiency phenotypes.

## Discussion

### sPLA2 activities modify PGRN-deficient phenotypes in the mouse brain

sPLA2-IIA is a member of a secreted PLA2 protein family (sPLA2), which catalyzes the hydrolysis of the sn-2 position of glycerophospholipids. It is an important mediator of inflammation through the generation of arachidonic acids (AAs) and other fatty acid signaling molecules [51, 57, 58]. The sPLA2 family consists of 11 members, each of which has distinct tissue-specific expression patterns and slight differences in substrate specificity [51, 57, 58]. In this study, we have identified sPLA2-IIA as a novel interactor for PGRN and shown that PGRN inhibits sPLA2-IIA activity in *vitro* (Fig. 6a and d). In addition, PGRN loss results in increased levels of sPLA2-IIA in the gut and elevated levels of prostaglandins and fatty acids in the serum (Fig. 6e and h). Among sPLA2 members, only sPLA2-IIA is known to be differentially expressed between FVB and B6, with a naturally occurring mutation and subsequent loss of sPLA2-IIA protein in the B6 background. We found that PGRN deficiency in the sPLA2-IIA expressing FVB mouse strain shows a much earlier onset and more severe phenotypes compared to the B6 background (Fig. 1-5). Additionally, AAV-mediated sPLA2-IIA overexpression in the cortex leads to enhanced phenotypes in PGRN-deficient B6 mice, indicating that increased sPLA2 activities in the brain exacerbate the phenotypes associated with PGRN loss. These findings led us to hypothesize that elevated sPLA2-IIA activity might contribute to the enhanced phenotype in the FVB strain with PGRN deficiency.

Elevated sPLA2 levels have been implicated in multiple inflammatory disorders [59]. The two sPLA2 inhibitors KH064 and LY333013 have been developed to treat sPLA2-associated diseases [59]. Remarkably, KH064 or LY333013 treatment significantly alleviates gliosis and lysosomal abnormalities in PGRN-deficient mouse brains in the FVB background (Fig. 7). While KH064 has been reported as a specific inhibitor of sPLA2-IIA [60], LY333013 is a broad inhibitor that targets several sPLA2 members, including sPLA2-V and sPLA2-X [61]. At this stage, we can not rule out the possibility that these two inhibitors might also target other sPLA2 family members or even other unrelated proteins to regulate inflammation and lysosomal activities in PGRN-deficient mice, since sPLA2-IIA level is low in the brain. Nevertheless, our results suggest that the two sPLA2 inhibitors might be a valid therapeutic approach for FTLD-*GRN*, and possibly also other neurodegenerative diseases, to restrict neuroinflammation [62]. It will be worth testing the effect of sPLA2 inhibitors in other mouse models of neuroinflammation and neurodegeneration in the future.

In humans, although polymorphisms in the *Pla2g2a* gene affecting sPLA2-IIA levels have been associated with metabolic disorders, such as coronary heart disease and type II diabetes [63–65], no genetic link between *Pla2g2a* and FTLD or other neurodegenerative diseases has been reported. In the mouse brain, we have failed to detect endogenous sPLA2-IIA signals by western blot (Fig. 6e) or immunostaining (data not shown). However, sPLA2-IIA protein was detected in the brain lysate when human sPLA2-IIA is expressed under its native promoter in the *Tg-hPla2g2a* mice (Fig. S6b). Pevious studies have shown that sPLA2-IIA is expressed by astrocytes [66, 67] and endothelial cells [68]. Secreted sPLA2-IIA has been shown to cause neuronal toxicity [67], stimulate phagocytosis, proliferation, and inflammatory responses of microglia-derived BV2 cells [69], and proliferation of an astrocytoma cell line [70]. sPLA2-IIA levels are increased after spinal cord injury, and sPLA2-IIA has a detrimental effect [71]. In addition, sPLA2-IIA mRNA is up-regulated in Alzheimer’s disease (AD) brains, and sPLA2-IIA-immunoreactive astrocytes are associated with amyloid β (Aβ)-containing plaques in AD [62]. Oligomeric Aβ1–42 and interleukin-1β (IL-1β) were shown to induce sPLA2-IIA mRNA expression [62]. Thus, although sPLA2-IIA levels are low in the brain, brain-expressed sPLA2-IIA might still be subject to regulation by PGRN to regulate brain inflammatory phenotypes, especially in humans. On the other hand, the highest level of sPLA2-IIA expression is found in the gut (Fig. 6e), and sPLA_2_-IIA can regulate gut microbiota, inflammation, and metabolism [54–56]. It also functions as an intestinal stem cell niche factor to regulate intestine homeostasis, inflammation, and cancer [72]. An increase in sPLA2-IIA levels in the duodenum was found under PGRN-deficient conditions (Fig. 6f). Alterations in the gut can affect brain pathology through many different mechanisms [73, 74]. Therefore, another possibility is that PGRN may regulate brain inflammation phenotypes by modulating sPLA2-IIA function and levels in the gut. Additionally, an increase in the levels of downstream products of sPLA2-IIA, prostaglandin, and free fatty acids is observed in the serum of PGRN-deficient mice (Fig. 6G), which could pass blood-brain barrier to affect brain phenotypes. Future studies are needed to pinpoint the actions of PGRN and sPLA2-IIA in the periphery versus in the brain.

### Connections between inflammation and lysosomal abnormalities

Intriguingly, sPLA2-IIA overexpression not only leads to glial inflammation but also lysosomal abnormalities and lipofuscin accumulation and sPLA2-IIA inhibitors rescue both lipofuscin accumulation and lysosomal abnormalities under PGRN deficient conditions (Figs. 7 and 8, and Fig. 9). Though the mechanisms by which sPLA2-IIA regulates lysosomal phenotypes remain to be investigated, many recent studies have established a close link between lysosomal dysfunction and inflammation. For example, lysosomal membrane permeability (LMP) often results in the release of lysosomal enzymes and substrates into the cytosol, which often leads to inflammasome activation [75–77]. In addition, the activation of TFE3/TFEB, the main transcriptional factor involved in lysosome biogenesis, drives the expression of not only lysosomal genes but also inflammation genes in several studies [78, 79]. Furthermore, substrate accumulation and lysosome stress can activate Stat3, which mediates the transcription of many genes involved in inflammatory responses [80]. However, whereas sPLA2-IIA is important for inflammatory responses by producing lipid mediators of inflammation, its role in the lysosomal compartment is completely unexplored. Interestingly, AAV9-mediated sPLA2-IIA overexpression in neurons in PGRN-deficient mouse brains triggers lipofuscin accumulation and lysosomal enlargement specifically in microglia (Fig. 9). Since sPLA2-IIA is a secreted enzyme, hydrolysis of lipids in extracellular vesicles or the extracellular leaflet of the plasma membrane may lead to the production of AAs and other inflammatory lipid mediators, such as prostaglandins [51, 57, 58]. The free fatty acids or other molecules produced by sPLA2-IIA could be uptaken by microglia to cause lysosomal abnormalities under PGRN-deficient conditions. sPLA2-IIA has also been shown to have catalytically independent functions, such as signaling through its receptors [51, 57, 58]. Thus, sPLA2-IIA might also affect microglia lysosomal activities through its signaling functions or other mechanisms.

### Mouse strain background modifies PGRN deficiency phenotypes

Previous studies have shown that the strain background of an animal model can significantly modify the phenotypes of neurodegenerative diseases, such as lysosomal storage disorders and AD [27, 81]. Investigating the phenotypes caused by PGRN deficiency in mice with different backgrounds is not only valuable for understanding the mechanisms underlying disease onset and progression but also important for developing proper disease mouse models. In this study, we found that mouse strain background modifies PGRN deficiency phenotypes. Specifically, PGRN deficiency in the FVB mouse strain leads to an earlier onset and more pronounced phenotypes, including gliosis, lysosomal defects, and TDP-43 pathology, as well as more significant alterations of gene and protein expression, compared to the B6 mouse strain (Fig. 1-Figs. 5 and 10, and Fig. 11). Our studies suggest that the *Grn*^*−/−*^ FVB mouse strain could be a valuable tool for studying the effects of PGRN loss, particularly for identifying early events involved in the development of PGRN deficiency phenotypes.

RNA-seq and proteomics analysis revealed significant differences between the two mouse strains, with PGRN loss leading to distinct alterations in gene and protein expression as well as in various biological pathways (Figs. 10 and 11). These analyses also identified common proteins regulated by PGRN, such as SCRB2, PPT1, SMPD1, and ASAH1, which have been shown to be affected by PGRN deficiency in previous studies [47, 82, 83]. Moreover, RNA-seq analysis suggested that lysosomal and immune pathways are upregulated by PGRN deficiency in both strains (Fig. 10h), while proteomics analysis suggests that inorganic transmembrane transport is upregulated in both strains (Fig. 11g), underscoring the role of PGRN in these pathways. Interestingly, both RNA-seq and proteomic analysis revealed the differential regulation of mitochondrial pathways by PGRN in the two strains (Figs. 10h and i and 11g and h).

### Connection between mitochondrial dysfunction and PGRN deficiency phenotypes

Mitochondrial dysfunction, including impaired ATP production, increased oxidative stress, and defective mitophagy, has been closely associated with neurodegenerative diseases, including LSDs and FTLD [84–87]. In our study, we observed that PGRN loss affects mitochondrial function differently in FVB and B6 strain backgrounds (Fig. 10h and i, and Fig. 11g and h). In response to PGRN loss, mitochondrial function is likely to be increased in the B6 mice, as evidenced by the upregulation of the oxidative phosphorylation/ATP metabolic pathway, which might exert a protective effect. In contrast, this compensatory response is absent in FVB mice, which might explain the more severe lysosomal and inflammatory phenotypes observed. These findings indicate that impaired mitochondrial function might enhance the PGRN deficiency phenotypes.

A key pathological feature observed in PGRN-deficient mouse brains is the accumulation of lipofuscin in microglia and neurons (Fig.S2). While the underlying mechanism behind this remains unclear, growing evidence points to a significant role of mitochondria in lipofuscin accumulation [88–92]. Intriguingly, several studies have demonstrated that PGRN regulates various aspects of mitochondrial functions. Disrupted mitochondrial integrity, impaired mitochondrial biogenesis, or defective mitophagy have been observed in conditions of PGRN deficiency [93–95]. Thus, it is plausible that mitochondrial dysfunction contributes to impaired lysosomal activity and lipofuscin accumulation in the PGRN-deficient mouse brain, which might lead to increased TDP-43 aggregation (Fig. 5) since the autophagy-lysosomal pathway is critical for TDP-43 turnover [96–98]. Moreover, mitochondrial defects may trigger inflammation [99, 100]. Interestingly, a recent study has demonstrated that PGRN deficiency leads to mitochondrial hyperfusion and bioenergetic deficits in mice, which activate complement C3a-C3aR signaling, subsequently triggering microglia activation [101]. Therefore, mitochondrial dysfunction might contribute to both lysosomal defects and enhanced inflammation observed under PGRN deficiency conditions, and future studies are needed to dissect the detailed mechanisms.

## Electronic supplementary material

Below is the link to the electronic supplementary material.

Supplementary Material 1

## Data Availability

Raw LC-MS/MS data for mouse brain proteomics are available through the MassIVE repository (Identifier: MSV000097392). Other data is provided within the manuscript or supplementary information files.
