# Supplementary material for: sPLA2-IIA modifies progranulin deficiency phenotypes in mouse models
Source: Mol Neurodegener. 2025 Jun 17;20:72. doi: 10.1186/s13024-025-00863-8 (PMC12175356; doi:10.1186/s13024-025-00863-8)

Supplementary Information:

Molecular pathways modifying progranulin deficiency phenotypes in mouse models

Cha Yang[1,3]*, Huan Du[1,3]*, Gwang Bin Lee[5], Masaaki Uematsu[2,3], Weiguo He[1,3], Etienne Doré[4], Weizhi Yu[2,3], Ethan J. Sanford[1,3], Jeremy M. Baskin[2,3], Eric Boilard[4], Marcus B. Smolka[1,3], Ling Hao[5] and Fenghua Hu[1,3#]

Supplementary Figures 1-8

Description of the Supplementary Datasets:

Dataset 1: List of the proteins identified in the SILAC-based proteomic screen

Dataset 2: List of the DEGs identified in the brain RNA-seq analysis

Dataset 3: List of the proteins identified in the brain proteomics analysis

Supplemental figures:

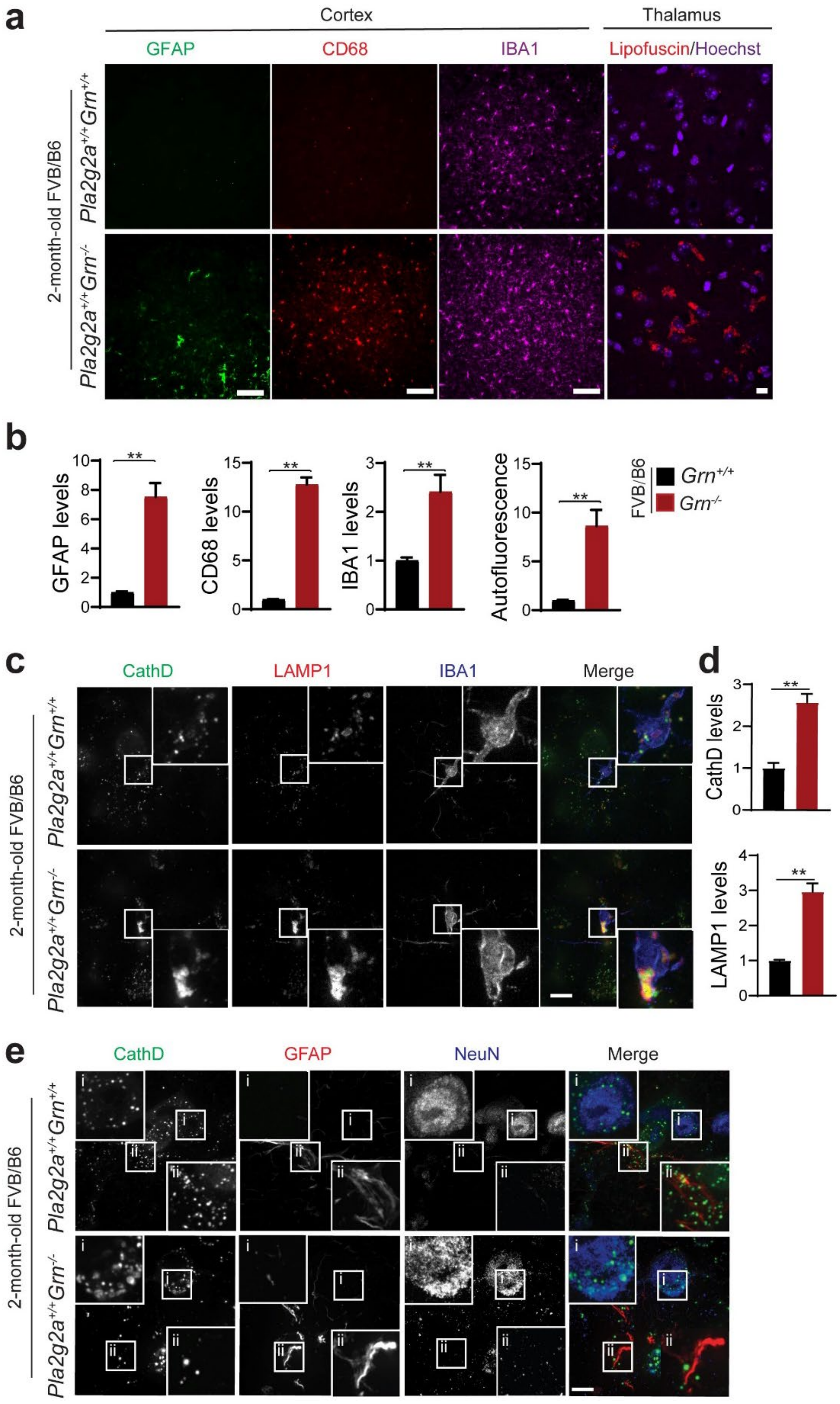

Fig. S1: PGRN deficiency in the sPLA2-IIA expressing background leads to early glial activation and lysosomal defects. (**a**) Immunostaining of GFAP, CD68, and IBA1 and auto-fluorescent lipofuscin signals in brain sections from 2-month-old *Pla2g2a*$^{+/+}$*Grn*$^{+/+}$ and *Pla2g2a*$^{+/+}$*Grn*$^{-/-}$ mice in the FVB/B6 hybrid background. Representative images from the cortex were shown for GFAP/CD68/IBA1 staining and images from the thalamus were shown for lipofuscin signals. Scale bar =100 µm (GFAP/CD68/IBA1); Scale bar =10 µm (lipofuscin). (**b**) Quantification of GFAP, CD68, and IBA1 levels and auto-fluorescent lipofuscin signals for the experiment in (A). Data are presented as mean ± SEM from three mice per group (n=3). **, p<0.01, Student's t-test. (**c, d**) Immunostaining of CathD, LAMP1, and IBA1 in brain sections from 2-month-old *Pla2g2a*$^{+/+}$*Grn*$^{+/+}$ and *Pla2g2a*$^{+/+}$*Grn*$^{-/-}$ mice. Representative images from the cortex were shown. CathD and LAMP1 signals in IBA1-positive microglia were quantified (d). Scale bar =10 µm. 10-12 cells per section were analyzed. 3 sections per mouse brain and 3 mouse brains were used for each genotype (n=3). **, p<0.01. (**e**) Immunostaining of CathD, GFAP, and NeuN in brain sections from 2-month-old *Pla2g2a*$^{+/+}$*Grn*$^{+/+}$ and *Pla2g2a*$^{+/+}$*Grn*$^{-/-}$ mice. Representative images from the cortex were shown. Similar results were obtained from 3 mouse brains. Scale bar = 10 µm.

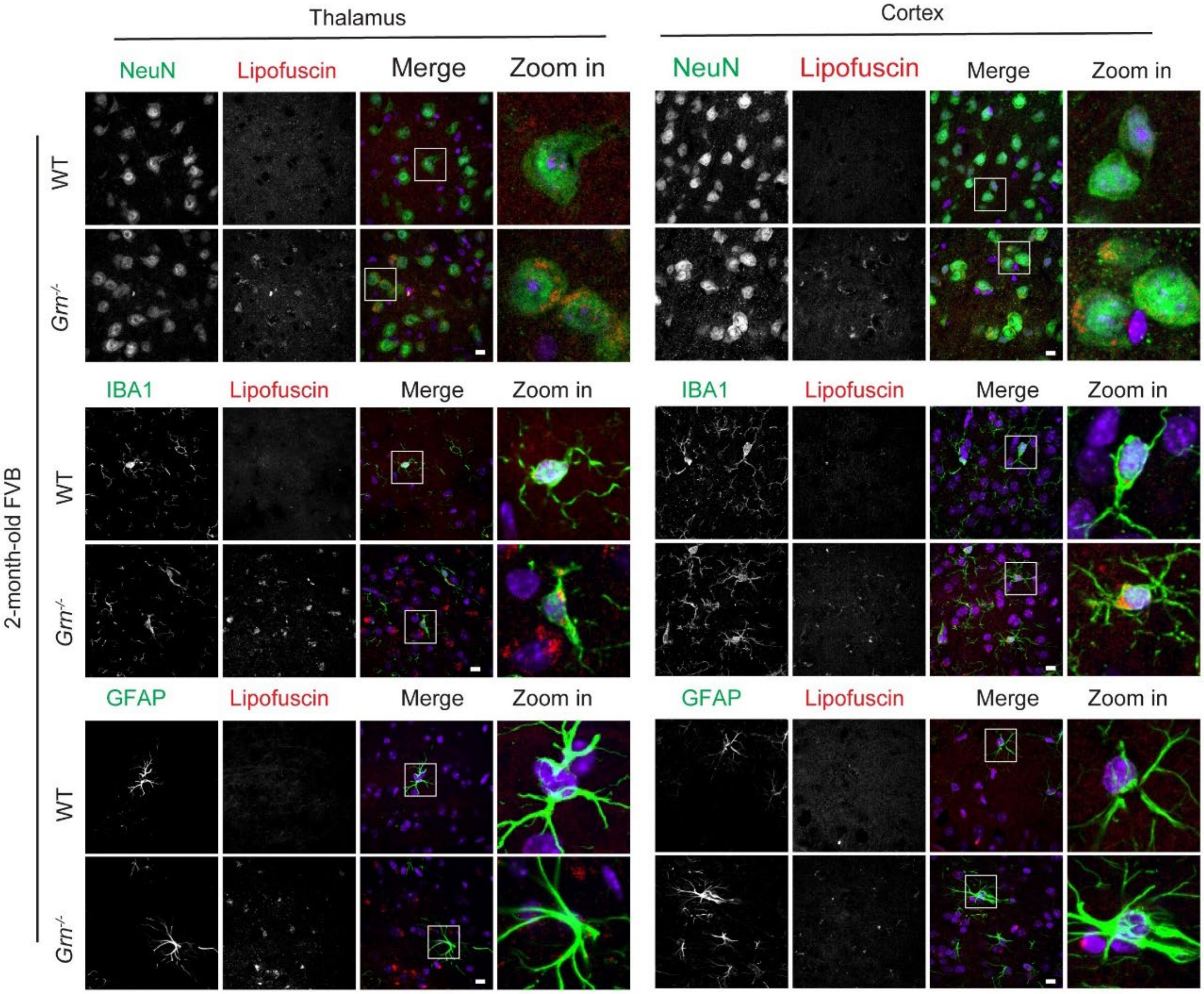

Fig. S2: Lipofuscin is accumulated in microglia and neurons in PGRN-deficient mouse brain. Colocalization of lipofuscin signals with NeuN, IBA1, and GFAP markers. Representative images from the cortex and thalamus of 2-month-old WT and *Grn*$^{-/-}$ mice in the FVB background were shown. Scale bar =10 µm.

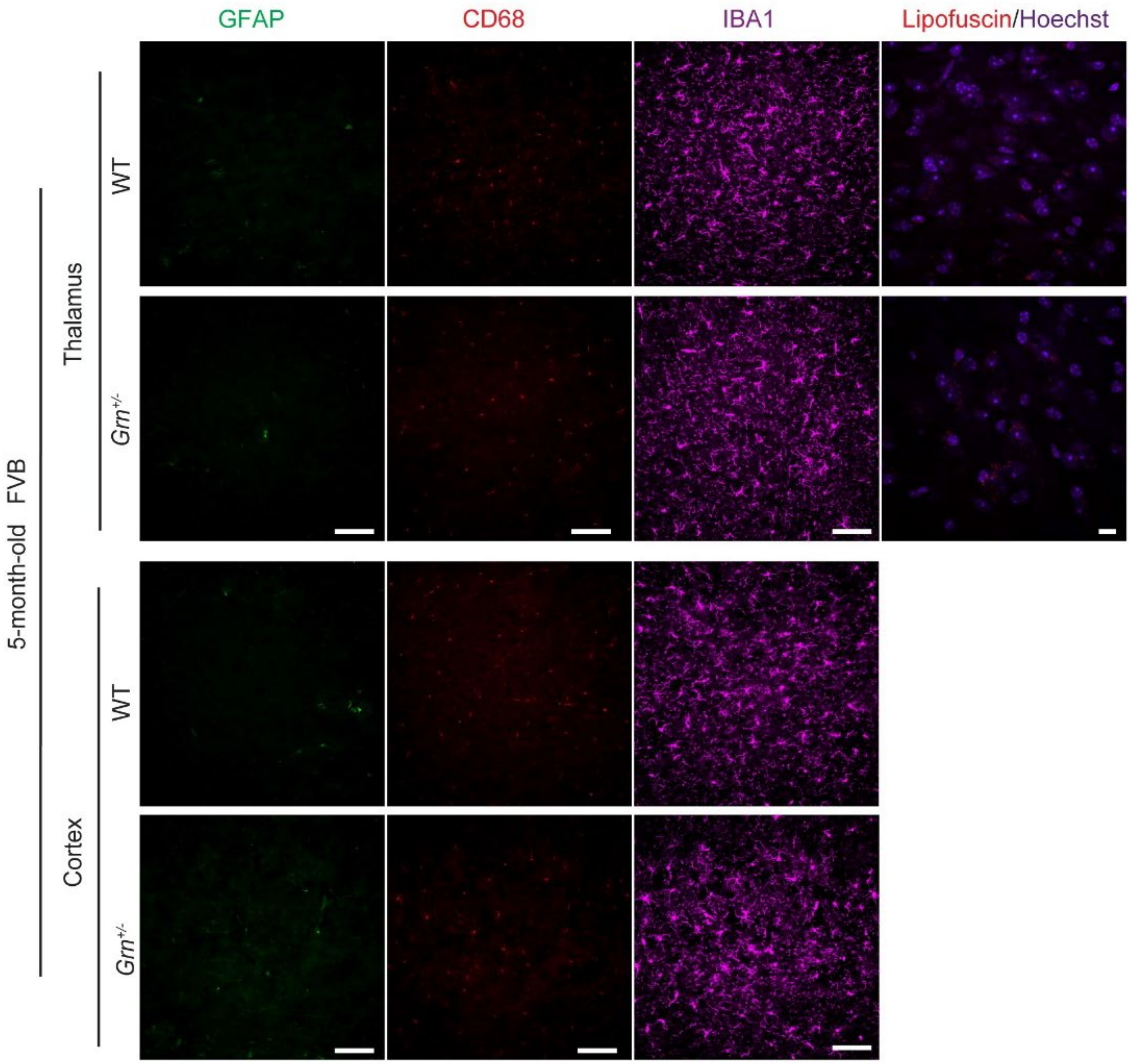

Fig. S3: Haploinsufficiency of PGRN does not cause gliosis and lipofuscin accumulation in the FVB mice. Immunostaining of GFAP, CD68, and IBA1 and auto-fluorescent lipofuscin signals in brain sections from 5-month-old WT and *Grn*$^{+/-}$ mice. Representative images from the thalamus and cortex were shown. Scale bar =100 µm (GFAP/CD68/IBA1); Scale bar =10 µm (lipofuscin).

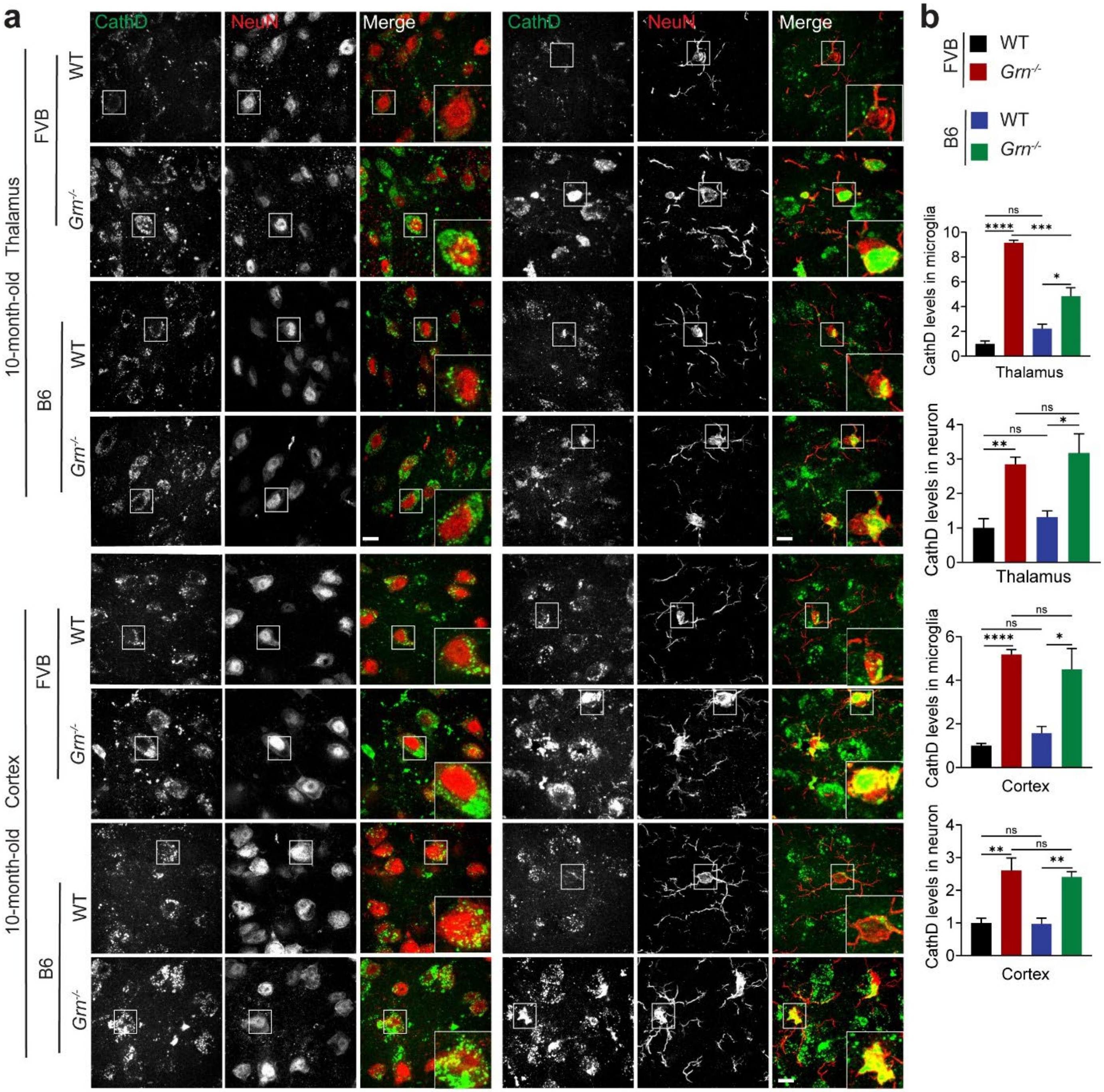

Fig. S4: Lysosomal changes in PGRN-deficient mice in FVB and B6 background. (**a, b**) Immunostaining of CathD, NeuN, and IBA1 in brain sections from 10-month-old WT and *Grn*$^{-/-}$ mice in the FVB and the B6 strain background. Representative images from the cortex and thalamus were shown. CathD signals in NeuN-positive neurons and IBA1-positive microglia were quantified. 3 sections per mouse brain and 3-4 mouse brains were used for each genotype. 25-40 microglia and 45-80 neurons per section were analyzed. Data are presented as mean ± SEM from 3-4 mice per group. n=3-4, *, $p<0.05$, **, $p<0.01$, ***, $p<0.001$. Student's t-test. Scale bar=10μm.

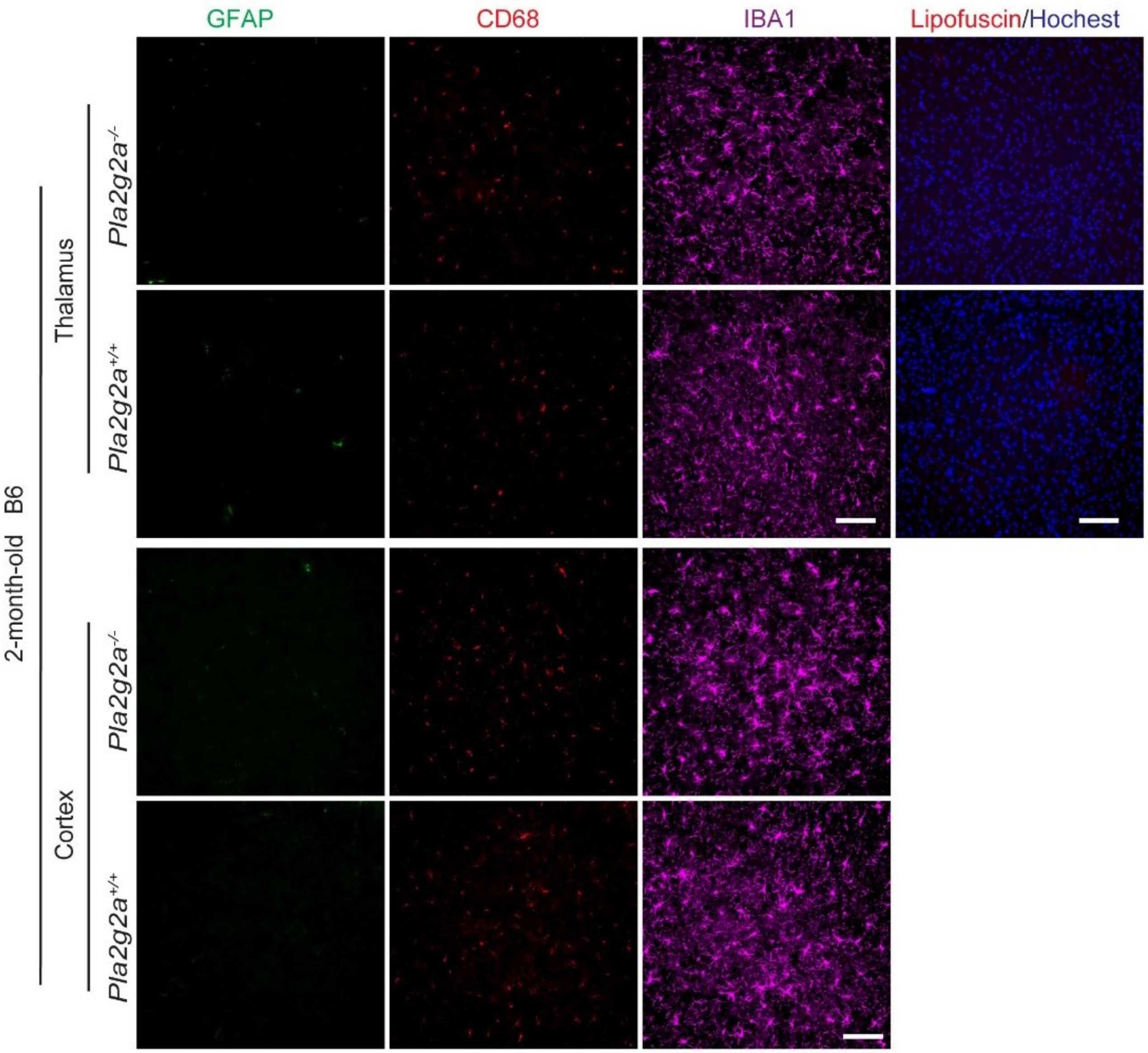

Fig. S5: Expression of mouse sPLA2-IIA in the B6 background does not cause glial activation or lipofuscin accumulation. Immunostaining of GFAP, CD68, and IBA1 and auto-fluorescent lipofuscin signals in brain sections from 2-month-old WT (*Pla2g2a*$^{-/-}$) and mouse sPLA2-IIA-expressing (*Pla2g2a*$^{+/+}$) mice in B6 background. Representative images from the cortex and thalamus were shown for GFAP/CD68/IBA1 staining and images from the thalamus were shown for lipofuscin signals. Scale bar =100 µm. Similar results were obtained from 4 mouse brains.

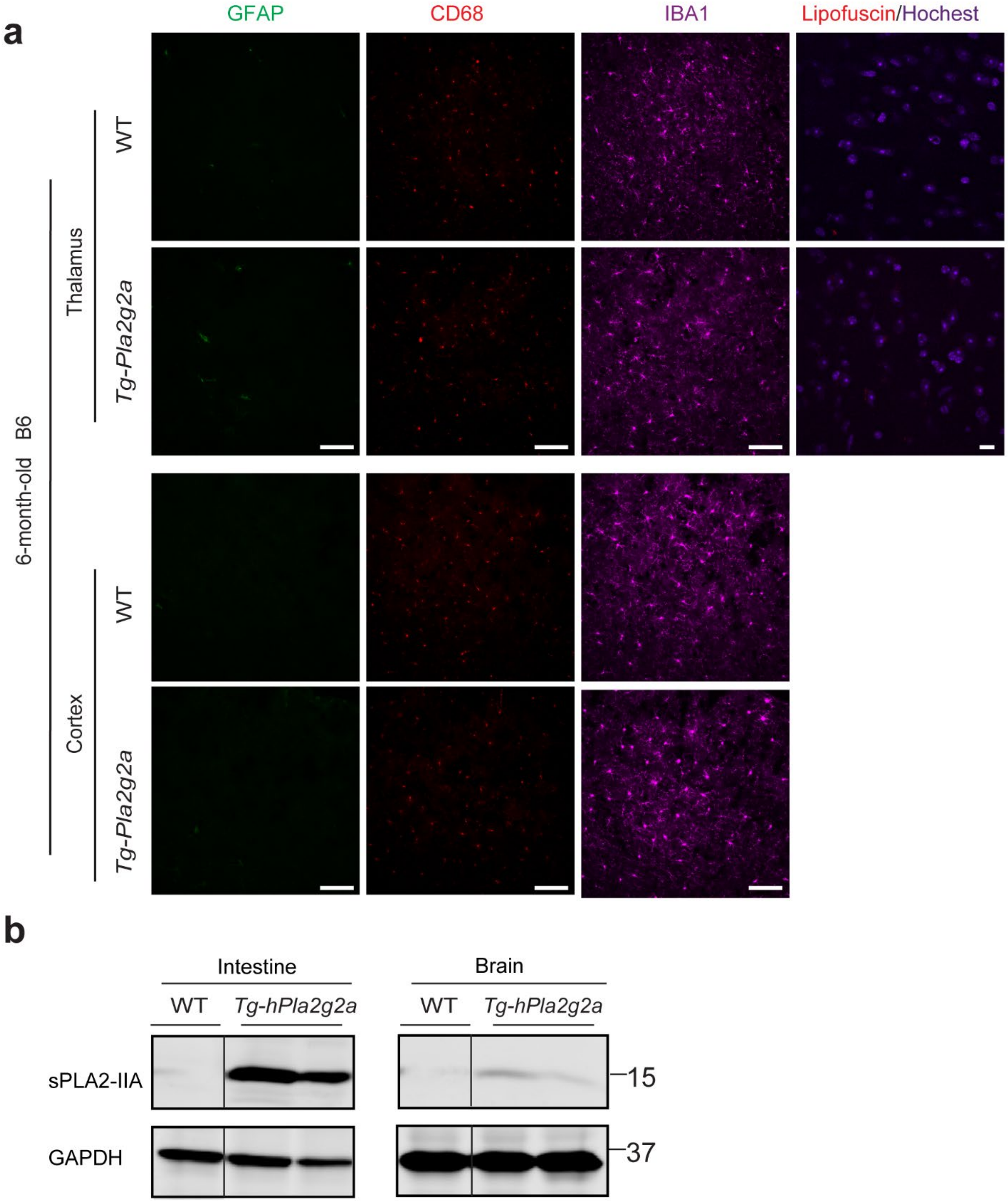

Fig. S6: Expression of human sPLA2-IIA in the B6 background does not cause glial activation or lipofuscin accumulation. (**a**) Immunostaining of GFAP, CD68, and IBA1 and auto-fluorescent lipofuscin signals in brain sections from 6-month-old WT and *Tg-hPla2g2a* mice. Representative images from the cortex and thalamus were shown for GFAP/CD68/IBA1 staining and images from the thalamus were shown for lipofuscin signals. Scale bar =100 μm (GFAP/CD68/IBA1); Scale bar =10 μm (lipofuscin). Similar results were obtained from 3 mouse brains. (**b**) Western blot analysis of human sPLA2-IIA in the intestine and whole brain lysates of the WT control and *Tg-hPla2g2a* mice.

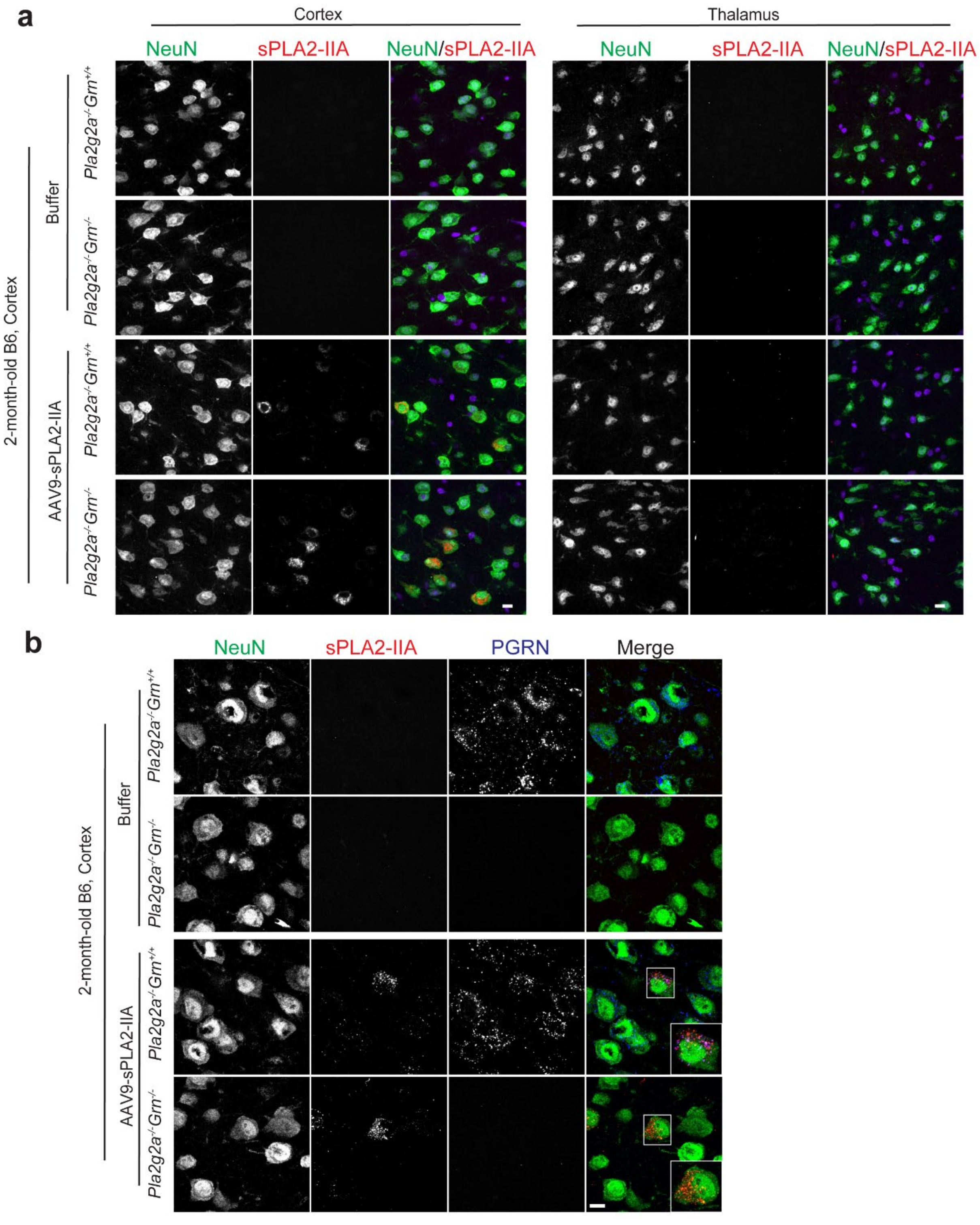

Fig. S7: The AA9-mediated sPLA2-IIA expression in WT and PGRN-deficient B6 mouse brain. B6 WT ($Pla2g2^{-/-}Grn^{+/+}$ and $Grn^{-/-}$ ($Pla2g2a^{-/-}$ $Grn^{-/-}$) pups at postnatal day (P0) were injected with AAV9-sPLA2-IIA or the buffer control and the mice were collected at 2-month-old. (**a**) AAV9-sPLA2-IIA is expressed in neurons in cortex. Brain sections were immunostained with anti-sPLA2-IIA and NeuN antibodies, and representative images from the cortex and thalamus region were shown (Scale bar=10 μm). (**b**) sPLA2-IIA colocalizes with PGRN in neurons. Brain sections were immunostained with anti-sPLA2-IIA, PGRN, and NeuN antibodies, and representative images from the cortex region were shown (Scale bar=10 μm).

Fig. S8: Uncropped WB images.

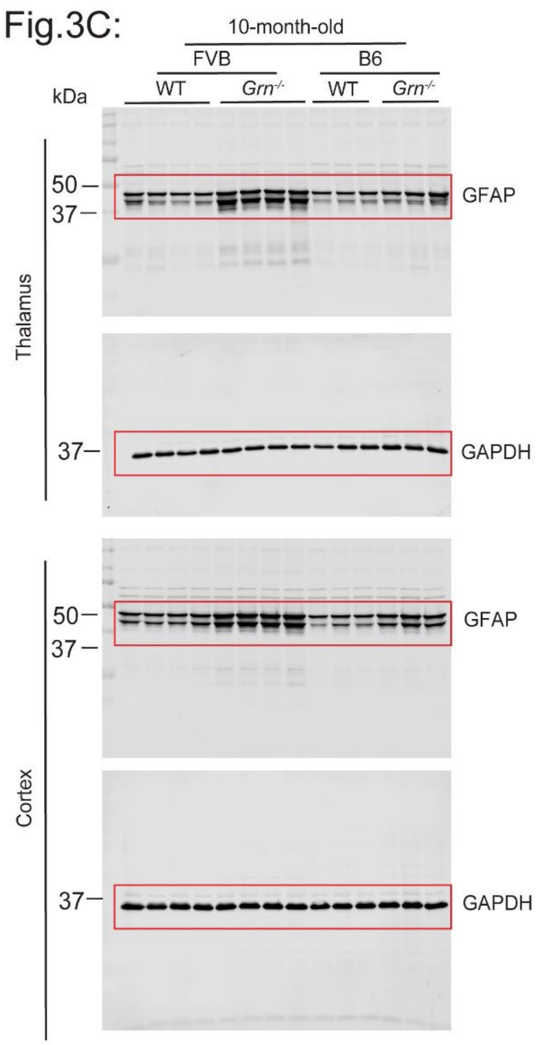

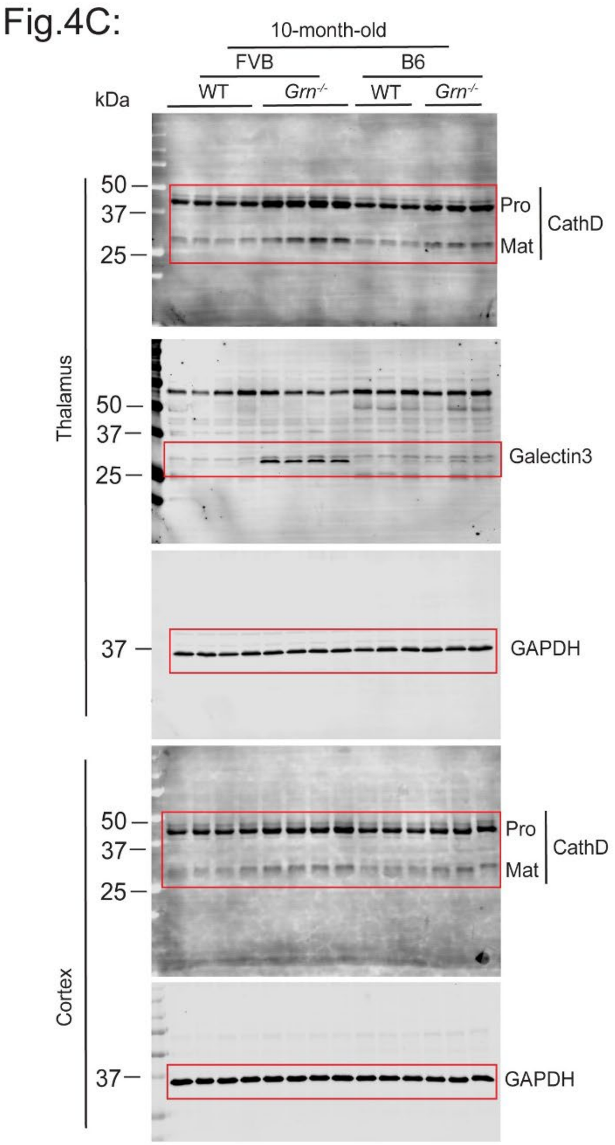

Fig.5A:

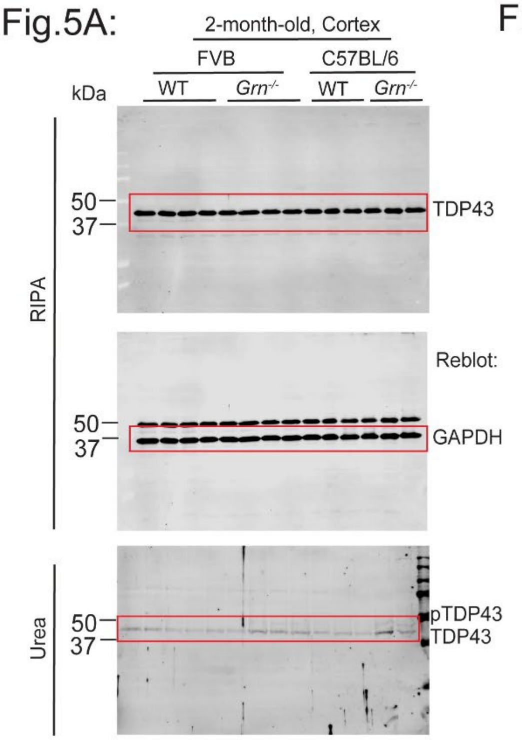

Fig.5B:

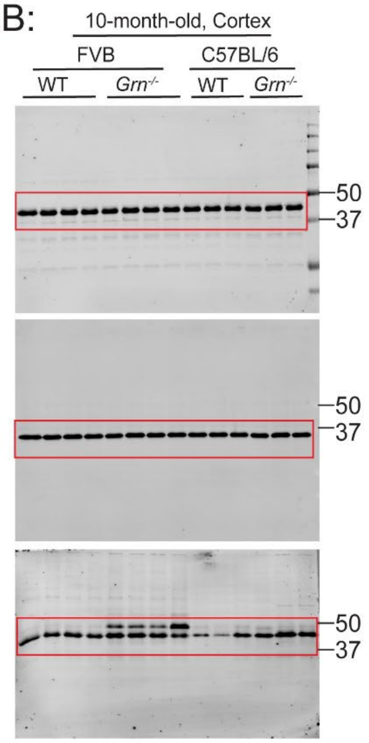

Fig.S6B:

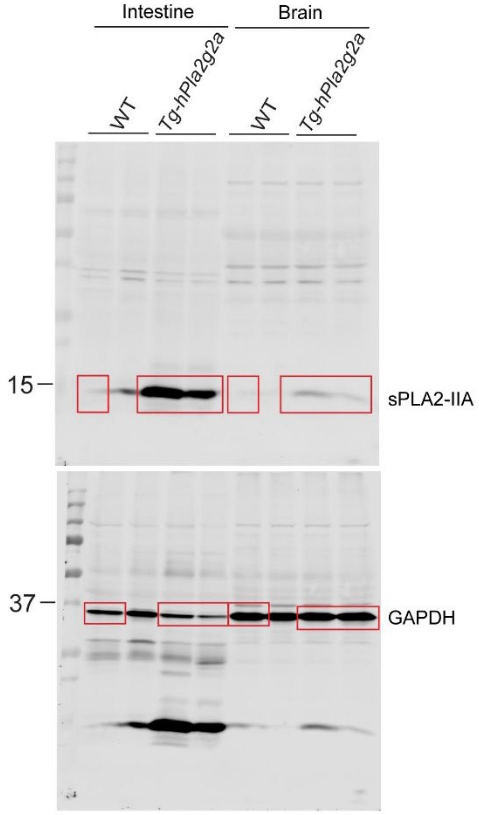

Fig.6B:

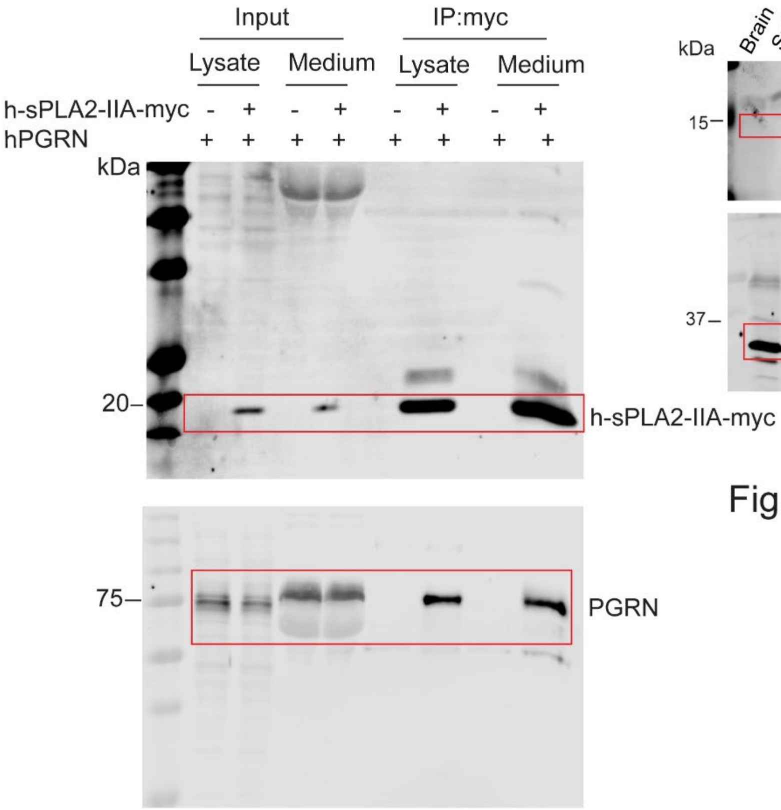

Fig.6E:

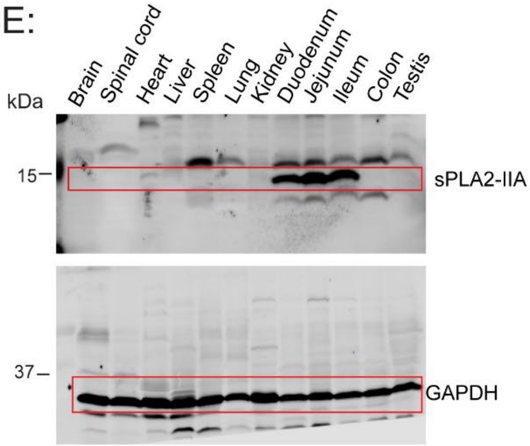

Fig.6F:

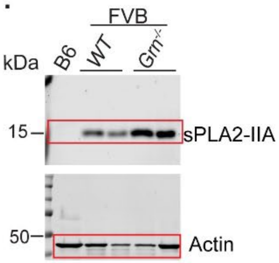

Fig.6C:

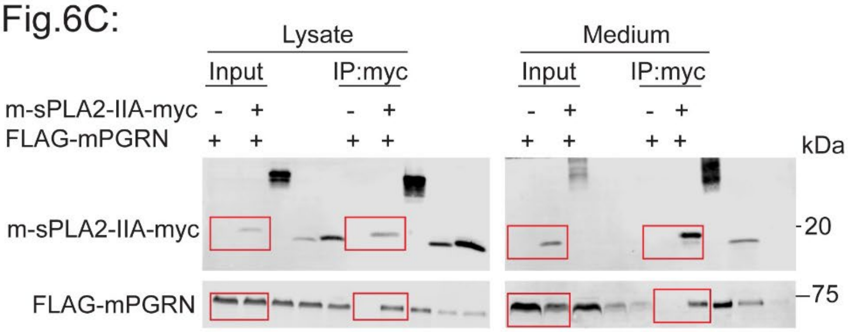

Supplement: Supplementary file 1 — Supplementary Material 1 [file 13024_2025_863_MOESM1_ESM.pdf]
